# *EHHADH* contributes to cisplatin resistance through regulation by tumor-suppressive microRNAs in bladder cancer

**DOI:** 10.1186/s12885-020-07717-0

**Published:** 2021-01-11

**Authors:** Shunsuke Okamura, Hirofumi Yoshino, Kazuki Kuroshima, Masafumi Tsuruda, Yoichi Osako, Takashi Sakaguchi, Masaya Yonemori, Yasutoshi Yamada, Shuichi Tatarano, Masayuki Nakagawa, Hideki Enokida

**Affiliations:** Department of Urology, Graduate School of Medical and Dental Sciences, Kagoshima University, 8-35-1 Sakuragaoka, Kagoshima, 890-8520 Japan

**Keywords:** Cisplatin resistance, Bladder cancer, *EHHADH*, *miR-486-5p*

## Abstract

**Background:**

Cisplatin-based chemotherapy is recommended as the primary treatment for advanced bladder cancer (BC) with unresectable or metastatic disease. However, the benefits are limited due to the acquisition of drug resistance. The mechanisms of resistance remain unclear. Although there are some reports that some molecules are associated with cisplatin resistance in advanced BC, those reports have not been fully investigated. Therefore, we undertook a new search for cisplatin resistance-related genes targeted by tumor suppressive microRNAs as well as genes that were downregulated in cisplatin-resistant BC cells and clinical BC tissues.

**Methods:**

First, we established cisplatin-resistant BOY and T24 BC cell lines (CDDP-R-BOY, CDDP-R-T24). Then, Next Generation Sequence analysis was performed with parental and cisplatin-resistant cell lines to search for the microRNAs responsible for cisplatin resistance. We conducted gain-of-function analysis of microRNAs and their effects on cisplatin resistance, and we searched target genes comprehensively using Next Generation mRNA sequences.

**Results:**

A total of 28 microRNAs were significantly downregulated in both CDDP-R-BOY and CDDP-R-T24. Among them, *miR-486-5p*, a tumor suppressor miRNA, was negatively correlated with the TNM classification of clinical BC samples in The Cancer Genome Atlas (TCGA) database. Transfection of *miRNA-486-5p* significantly inhibited cancer cell proliferation, migration, and invasion, and also improved the cells’ resistance to cisplatin. Among the genes targeted by *miRNA-486-5p*, we focused on enoyl-CoA, hydratase/3-hydroxyacyl CoA dehydrogenase (*EHHADH*), which is involved in the degradation of fatty acids. *EHHADH* was directly regulated by *miRNA-486-5p* as determined by a dual-luciferase reporter assay. Loss-of-function study using *EHHADH* si-RNA showed significant inhibitions of cell proliferation, migration, invasion and the recovery of cisplatin sensitivity.

**Conclusion:**

Identification of *EHHADH* as a target of *miRNA-486-5p* provides novel insights into the potential mechanisms of cisplatin resistance in BC.

## Background

Bladder cancer (BC) can be roughly classified into non-muscle-invasive BC (NMIBC) and muscle-invasive BC (MIBC). Approximately 50% of MIBC patients develop metastasis within 2 years, and the 5-year survival rate remains under 50% [1]. Advanced BC patients are generally treated with cisplatin-based combination chemotherapy as neoadjuvant and adjuvant therapy [2]. Although the overall survival rate (OS) has been extended by cisplatin, the median OS is only about 14 months after cisplatin based-chemotherapy [3, 4]. Because the molecular mechanisms of resistance to cisplatin in BC remain unclear, studies of the mechanism and novel prognostic markers to overcome cisplatin resistance are needed to improve outcomes in patients with BC.

Pharmacologically, cisplatin goes through hydrolysis and avidly binds DNA through the N7-sites of either a guanine or adenine base. Crosslinks and damage to DNA result, and apoptosis is induced [5, 6]. However, cancer cells can be initially resistant to cisplatin or they can acquire resistance [7]. For example, the high-affinity copper transporter (*CTR1*) is involved in the intracellular uptake of cisplatin. Knockdown of *CTR1* reduces the intracellular accumulation of cisplatin and induces cisplatin resistance [8]. Moreover, the epithelial mesenchymal transition (EMT) induces cisplatin resistance [9, 10]. In spite of intense study of cisplatin resistance, it has not yet been overcome.

MicroRNAs are endogenous small non-coding RNA molecules (19 ~ 22 bases in length) that regulate the expression of protein-coding/protein non-coding genes [11]. Our previous studies showed that microRNAs play many roles in cancer, including cancer cell progression, migration and invasion, and some microRNAs have significant roles in human oncogenesis [12, 13]. microRNAs are involved in cisplatin resistance in lung, gastric tissue, colon and ovarian cancers [14, 15]. Yang et al. reported that the transfection of *miRNA-214* into ovarian cancer cell lines suppressed *PTEN,* activated protein kinase *AKT* and led to cisplatin resistance [16]. In addition, Zhu et al. determined that *miRNA-181b* was downregulated in cisplatin-resistant lung cancer lines, and that *miRNA-181b* modulated cisplatin resistance by targeting the anti-apoptotic gene *BCL2* [17]. In BC, the relationships between microRNAs and cisplatin resistance are poorly understood. Therefore, this study focused on the effects of microRNAs on cisplatin resistance.

In order to elucidate the mechanism of cisplatin resistance, we established cisplatin-resistant BC cell lines (CDDP-R-BOY and CDDP-R-T24). Then, small-RNA sequence analyses were performed with the parental and resistant cell lines to search for the microRNAs associated with cisplatin resistance. The candidate microRNA was transduced into cisplatin-resistant cell lines for functional analysis. Next, we searched for target genes using RNA next-generation sequence analysis. We also performed loss of function studies to assess its target gene.

## Methods

### BC cell lines and culture

**Fig. 1:**
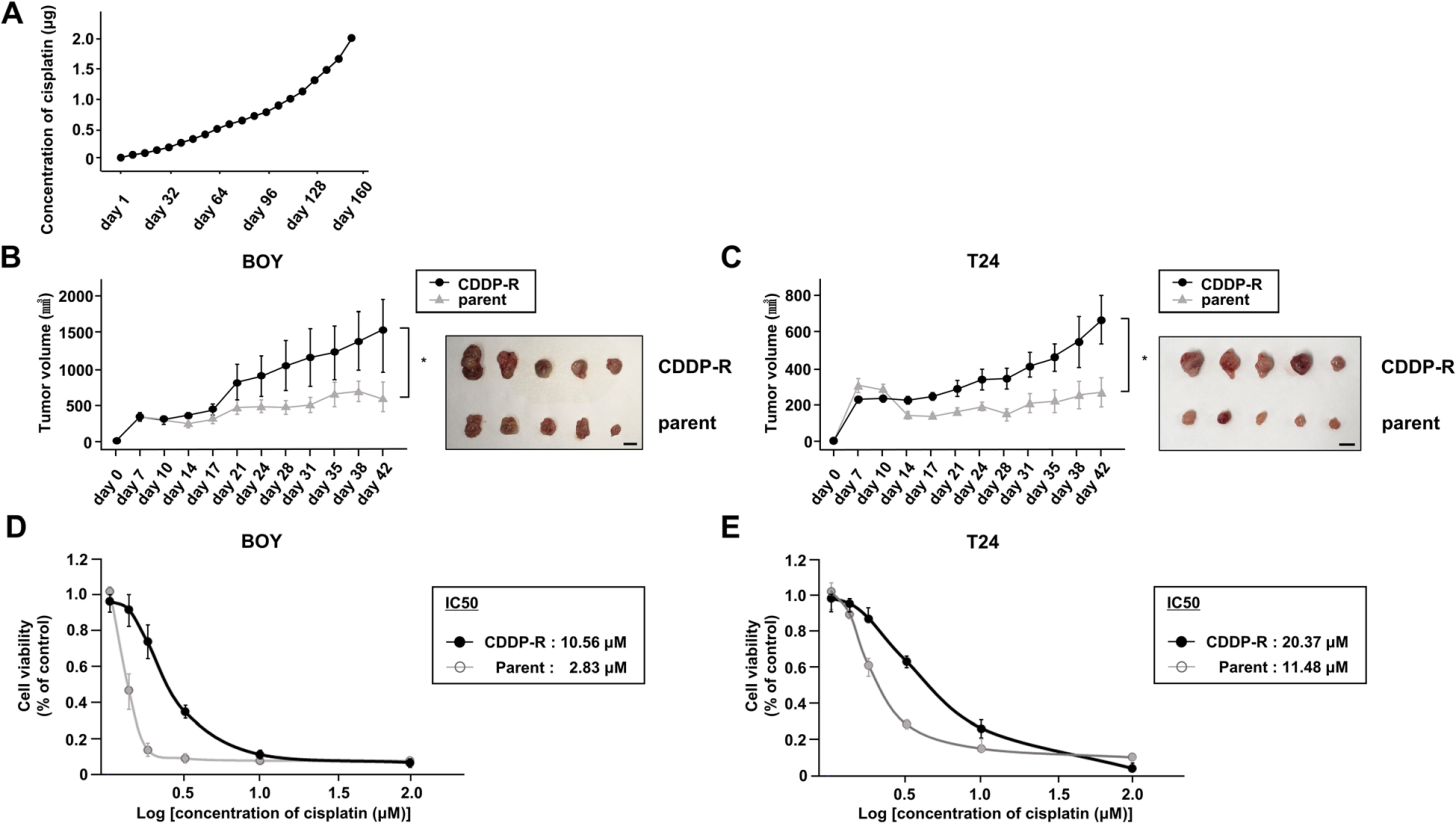
Establishment of cisplatin-resistant BC cells. **a** BC cells (BOY and T24) were cultured with serial concentrations of cisplatin from 0.01 to 2.0 μg/mL for 6 months. Time course comparing tumor volumes of (**b**) parental BOY/CDDP-R-BOY and (**c**) parental T24/CDDP-R-T24 after subcutaneous injection of cisplatin (4 mg/kg/mouse/week, *n* = 5 mice per group) *, *P* < 0.05. Scale bar: 10 mm. Calculated IC50 of (**d**) parental BOY/CDDP-R-BOY and (**e**) parental T24/CDDP-R-T24

We used 2 human BC cell lines: BOY was established in our laboratory from a 66-year-old Asian male patient, who was diagnosed with BC stage IV with many lung metastases. T24 was obtained from the American Type Culture Collection (Manassas, VA, USA). Generation of CDDP-R-BOY and CDDP-R-T24 is described in Fig. 1a. These cell lines were cultured in minimum Essential Medium Eagle (MEME) containing 50 mL of 10% fetal bovine serum (FBS), 50 μg/mL streptomycin, and 50 U/mL penicillin in a humidified atmosphere of 95% air/5% CO_2_ at 37 °C. To establish CDDP-R BC cell lines, we cultured BC cell lines with serial concentrations of cisplatin from 0.01 to 2.0 μg/mL for 6 months. The cells were cultured in 10 mL of medium for 24–36 h containing 1 mL of cisplatin that had been adjusted to 10-times the target concentration.

### Validation of the CDDP-R BC cell lines in vivo

The animal research described here was conducted in accordance with the regulations covering animal experiments at Kagoshima University, and the plan was approved by the Animal Experiment Committee at Kagoshima University (MD18094). We used 10 female athymic nude mice (BALB/c-nu/nu), aged 5 weeks, that were purchased from Charles River Laboratories (Yokohama, Japan). Sample size was determined by the guidelines for the welfare and use of animals in cancer research [18]. Mice were maintained in a standard laboratory environment (12-h day/night cycle, temperature, 25 °C), using rectangular mouse cages (225 × 338 × 140mm). Two or 3 mice were housed per cage, and a total of 4 cages were used. The cages were lined with sawdust to ensure water absorption and flexibility, and mice had continuous access to water and a standard diet (CLEA Rodent Diet CL-2) and were cleaned once a week. We used CDDP-resistant cells (CDDP-R-BOY and CDDP-R-T24) and their parental cells (BOY, T24) as controls. Four BC cell lines (BOY, CDDP-R-BOY, T24, and CDDP-R-T24) were adjusted to 1 × 10^7^ /mL, and 100 μL of the BC cells and 100 μL of Matrigel (BD Biosciences) were mixed. We subcutaneously injected parental cells (BOY, T24) into the right flanks and CDDP-R cells (CDDP-R-BOY, CDDP-R-T24) into the left flanks in a volume of 200 μL (5 mice per group). The measurement of mouse body weight and tumor size was started 7 days after the inoculation and conducted twice a week. Tumor dimensions were measured with calipers and sizes were calculated by the following formula: v = (length × width^2^) × (π/ 6). The administration of cisplatin (4 mg/kg, 5 times/week) [19] also started 7 days after the inoculation. At the endpoint of the experiment (6 weeks after the inoculation) all mice were euthanized with high concentrations (90–100%) of isoflurane and the tumor sizes were evaluated. There were no criteria used for including or excluding animals during the experiment. There was no exclusion of any experimental units or any data points for any experimental group. No adverse events were observed. The statistical significance of the difference of tumor size between the groups was analyzed with the Mann-Whitney U test using Expert StatView software, version 5.0 (Cary, NC, USA). *P* values less than 0.05 were accepted as statistically significant. There were no confounders in the animal experiments. All animal experiments were conduced by SO, HY, KK, and MT.

### IC50 determination

For determination of the IC50 value, cells were seeded into 96-well plates at a density of 2000 cells per well in triplicate and treated with a series of dilute concentrations of cisplatin. After 96 h of incubation, cell proliferation was measured using the XTT assay method according to the manufacturer’s instructions. A probit regression model was used to calculate the IC50 value using ImageJ (imagej.nih.gov) software.

### Transfection with mature miRNA and small interfering RNA (siRNA)

BC cells were transfected with the Lipofectamine RNAiMAX transfection reagent (Thermo Fisher Scientific) and Opti-MEM (Thermo Fisher Scientific) with 10 nM miRNA and siRNAs as previously reported [20]. Mature microRNA (hsa-*miR-486-5p;* product ID: PM10546) and negative-control microRNA (negative control miRNA; product ID: AM 17111) were used in gain-of-function experiments. *EHHADH* si-RNA (cat No. HSS105529 and HSS105531) and negative-control si-RNA (D-001810-10) were used in loss-of-function experiments.

### MicroRNA and mRNA sequence analysis

To search for the microRNAs associated with cisplatin resistance, total RNAs extracted from BOY, CDDP-R-BOY, T24, and CDDP-R-T24 cell lines were subjected to microRNA sequencing, performed by RIKEN GENESIS CO., LTD., Tokyo, Japan. We compared parental and CDDP-R cell lines (BOY vs CDDP-R-BOY, T24 vs CDDP-R-T24), and selected miRNAs with significantly downregulated expression in the CDDP-R cell lines (fold-change < − 1.0). mRNA sequence analysis was performed by RIKEN GENESIS CO., LTD. to identify the target mRNA of *miRNA-486-5p*. For the samples, a TruSeq Stranded mRNA Library Prep Kit was used to create libraries, and Illumina Inc’s flow cell was used for sequencing. The valid read length was 150 bp, and the analysis was performed using a Multiplex method. Candidate target genes were significantly downregulated after transfection with *miRNA-486-5p* compared with control microRNA (fold-change < − 1.0) in CDDP-R-BOY and CDDP-R-T24.

### In silico analysis

In order to evaluate the clinical relevance of our findings, a TCGA cohort database of 413 patients with BLCA was used. This study follows the criteria for the publication guidelines provided by TCGA. Kaplan-Meier analysis was used to analyze overall survival (OS) using data in the OncoLnc dataset (http://www.oncolnc.org/). To search for the miRNAs associated with cisplatin resistance, we identified miRNAs that were lower in expression in CDDP-R cells compared to parental cells in both BOY and T24, and had been reported as tumor suppressor genes. To identify possible target genes of *miRNA-486-5p*, we extracted genes that were reduced by transfection of miR486-5p in mRNA sequence analysis with genes that may be targeted by *miRNA-486-5p* based on TargetScan database Release 7.1 (http://www.targetscan.org).

### RNA extraction and RT-qPCR

To quantify the expression of *miRNA-486-5p*, we used Stem-loop RT-PCR (TaqMan MicroRNA Assays; P/N: 4427975 for *miR-486-5p*: Applied Biosystems) according to previously published conditions [20]. *RNU48* (P/N: 001006; Applied Biosystems) was used as the internal control. With regard to *EHHADH,* we applied a SYBR-green quantitative PCR-based array approach. The primer set used for determination of *EHHADH* mRNA expression level was as follows: forward, 5′-AAACTCAGACCCGGTTGAAGA-3′ and reverse, 5′-TTGCAGAGTCTACGGGATTCT − 3′. For glucuronidase β (*GUSB*; internal control), the set was as follows: forward primer, 5′-CGTCCCACCTAGAATCTGCT-3′ and reverse primer, 5′-TTGCTCACAAAGGTCACAGG − 3′. The specificity of amplification was monitored using the dissociation curve of the amplified product.

### Western blotting

To prepare total protein lysates, we used NuPAGE LDS Sample Buffer (Invitrogen; Thermo Fisher Scientific). Immunoblotting was carried out with diluted anti-EHHADH (1:500; cat. no. 13412–1-AP; Proteintech Group, Inc., Chicago, IL, USA), anti-cleaved PARP antibodies (1:750, #5625; Cell Signaling Technology), PARP antibodies (1:750, #9532; Cell Signaling Technology) and anti-β-actin antibodies (1:5000; cat. no. bs-0061R; Bioss, Beijing, China). The secondary antibodies were peroxidase-labeled anti-rabbit IgG (1:5000; cat. no. 7074S; Cell Signaling Technology, Inc.) or anti-mouse IgG (1:5000; cat. no. 7074S; Cell Signaling Technology, Inc.). The protein levels were evaluated using ImageJ software (ver. 1.48; http://rsbweb.nih.gov/ij/index.html) as described previously [21, 22].

### Cell proliferation, migration, invasion assays, and apoptosis assays

To evaluate cell proliferation, we used XTT assays. T24 and BOY cells were seeded in 96-well plates with 2 × 10^3^ cells/well with 100 μL of medium containing of 10% fetal bovine serum (FBS). We determined the extent of cell proliferation 96 h after seeding with a Cell Proliferation Kit II (Roche Diagnostics GmbH, Mannheim, Germany) as described previously. When using cisplatin, we added 10 μL adjusted to 10-times the target concentration. Wound healing assays were used for cell migration activity. Cells (2 × 10^5^ per well) were plated in 6-well plates, and after 48 h of incubation, the cell monolayer was scraped using a P-20 micropipette tip. The initial gap length (0 h) and the residual gap length 24 h after wounding were calculated from photomicrographs. For cell invasion assays, we used modified Boyden chambers consisting of Matrigel-coated Transwell membrane filter inserts with 8 μM pores in 24-well tissue culture plates (BD Biosciences, San Jose, CA, USA). The cells that had passed through the pores and attached to the surface of the chamber were counted from photomicrographs. For apoptosis assays, double staining with FITC-Annexin V and propidium iodide was performed using a FITC Annexin V Apoptosis Detection Kit (BD Biosciences, Bedford, MA, USA) by flow cytometry (CytoFLEX Analyzer; Beckman Coulter, Brea, CA, USA). We classified cells in 4 categories: viable cells, dead cells, early apoptotic cells or apoptotic cells using Summit 4.3 software (Beckman Coulter). The sums of the percentages of early apoptotic and apoptotic cells were compared. The cells treated with 2 μg/mL cycloheximide were used as a positive control.

### Plasmid construction and dual-luciferase reporter assays

Partial wild-type (WT) sequences of the 3′-UTR of *EHHADH* or those with a deleted *miRNA-486-5p* target site were inserted between the XhoI and PmeI restriction sites in the 3′-UTR of the hRluc gene in the psiCHECK-2 vector (C8021; Promega, Madison, WI, USA). CDDP-R-BOY and CDDP-R-T24 cells were transfected with 50 ng of vector and 10 nM *miRNA-486-5p*. According to the manufacturer’s protocol (E1960; Promega), the activities of firefly and *Renilla* luciferases in cell lysates were determined with a dual luciferase assay system.

## Results

### Establishment of cisplatin-resistant BC cell lines

First, we established cisplatin-resistant BC cell lines (CDDP-R-BOY, CDDP-R-T24). BC cells (BOY and T24) were cultured with serial concentrations of cisplatin from 0.01 to 2.0 μg/mL (Fig. 1a). The cells grew well in the presence of 2.0 μg/mL cisplatin. CDDP-R cells were continuously exposed to cisplatin for the maintenance of resistance [23, 24]. There was no significant difference in cell proliferation between the parental cells and CDDP-R cells in vitro (Supplementary Figure 1a). To validate drug resistance in vivo, we subcutaneously injected parental cells into the right flanks and CDDP-R cells into the left flanks of nude mice. The mice were treated with cisplatin intraperitoneally every other week [19]. The tumor growth of parental xenografts (BOY and T24) was reduced by intraperitoneal administration of cisplatin. In contrast, the CDDP-R tumors (CDDP-R-BOY and CDDP-R-T24) were not suppressed, reflecting their resistance to cisplatin (Fig. 1b, c). To determine the extent of cisplatin resistance, we calculated the IC50 value. In the case of BOY, the IC50 of CDDP-R-BOY was 5-times greater than the IC50 concentration inhibiting BOY (BOY IC50: 2.83 μM, CDDP-R-BOY IC50: 10.56 μM); for CDDP-R-T24, it was twice that of T24 (T24 IC50: 11.48 μM, CDDP-R-T24 IC50: 20.37 μM) (Fig. 1d, e).

### Expression levels of *miRNA-486-5p* in CDDP-R-BC cell lines and BC specimens

**Fig. 2:**
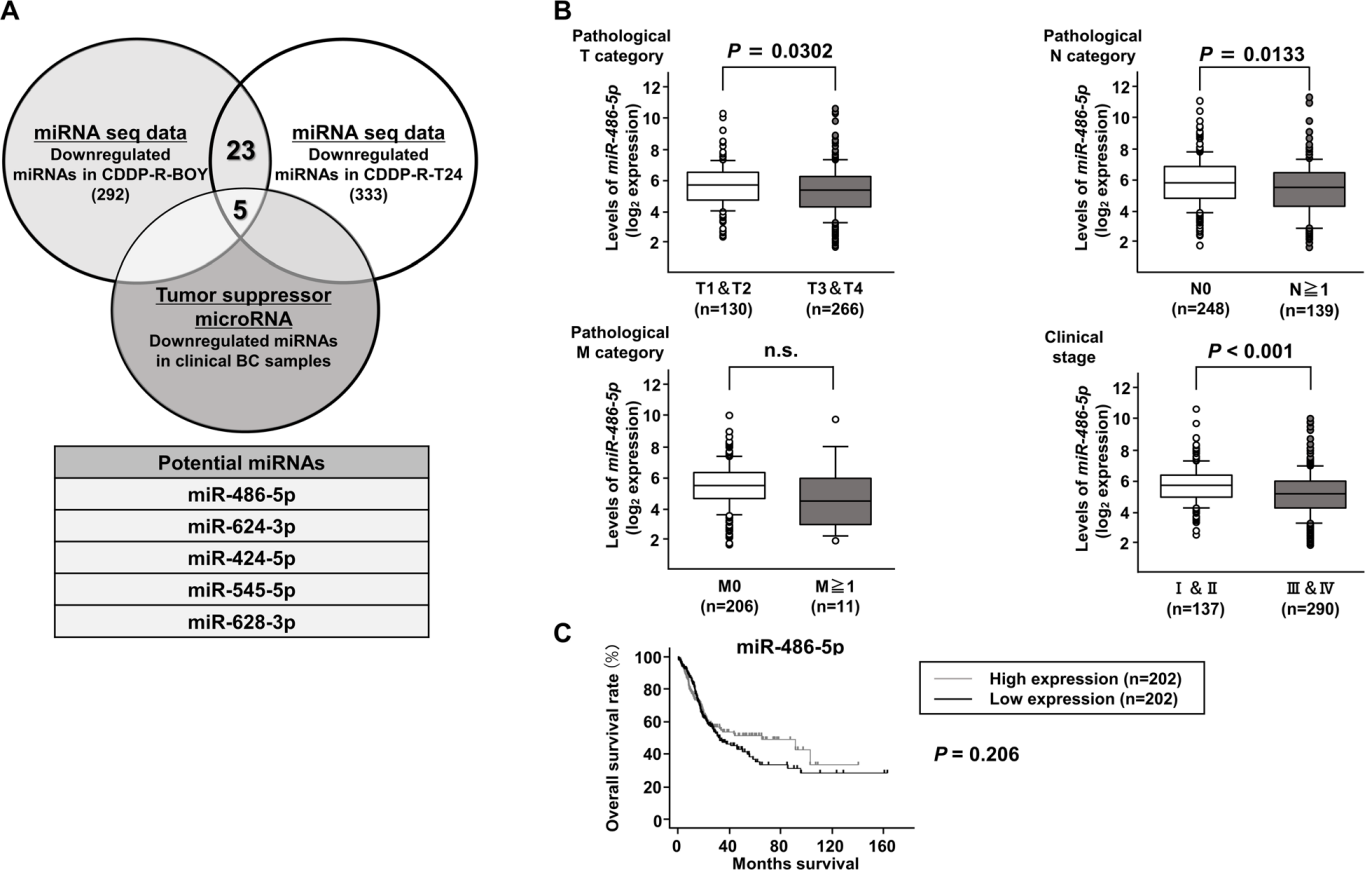
Expression levels of *miRNA-486-5p* in the CDDP-R-BC cell line and BC specimens. **a** Venn diagram of mRNA sequences and in silico analyses indicated 5 putative candidate miRNAs. **b** Among the BLCA cohort of TCGA, there were obvious negative correlations among expression levels of *miRNA-486-5p* and pathological T, N, and stage categories. **c** Kaplan-Meier analysis using the OncoLnc dataset revealed that there was a tendency for the high *miRNA-486-5p* expression group to have a lower OS than the low *miRNA-486-5p* expression group, but it did not reach significance (*P* = 0.206)

We performed microRNA sequence analysis of the parental and resistant cell lines to search for the miRNAs associated with cisplatin resistance. A total of 28 microRNAs were downregulated in both CDDP-R-BOY and CDDP-R-T24 cell lines. We searched for tumor-suppressive microRNAs, comparing BC and normal bladder epithelia by using a dataset reported by Itesako et al. [25]. Ultimately, 5 microRNAs (*miRNA-486-5p, miRNA-624-3p, miRNA-424-5p, miRNA-545-5p* and *miR-628-3p*) were identified as candidates (Fig. 2a). Among the bladder urothelial carcinoma (BLCA) cohort in TCGA, *miRNA-486-5p* and *miRNA-545-5p* showed significant difference between pathological category T1/2 vs T3/4. On the other hand, Kaplan-Meier analysis showed that overall survival (OS) exhibited no significant difference between the high expression group and the low expression group in *miRNA-486-5p* and *miRNA-545-5p* (Fig. 2c, Supplementary Figure 2b). Because *miRNA-545-5p* transfection did not suppress cell proliferation in CDDP-R BC cells (data not shown) and there was no correlations between miRNA-545-5p and the target gene (Supplementary Figure 2c), we focused on *miRNA-486-5p* as a strong candidate tumor suppressor that could overcome cisplatin resistance in this study.

### Effects of *miRNA-486-5p* restoration on cell proliferation, migration, invasion and apoptosis in CDDP-R-BC cell lines

**Fig. 3:**
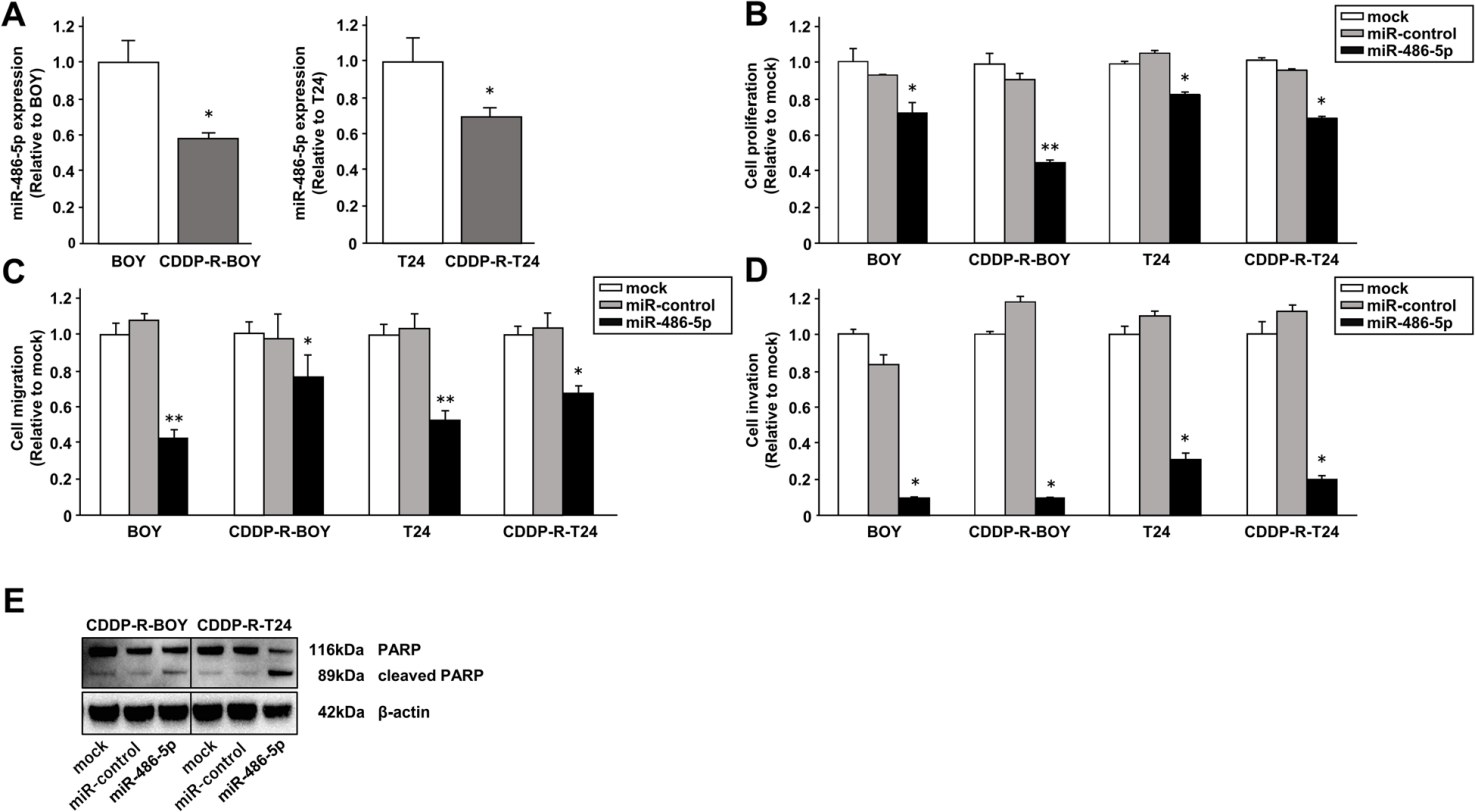
Effects of *miRNA-486-5p re*storation on cell proliferation, migration, invasion and apoptosis in CDDP-R-BC cell lines. **a** Expression levels of *miRNA-486-5p* determined by qRT-PCR were significantly lower in cisplatin-resistant BC cell lines than in parental BC cell lines. *, *P* < 0.05. **b** Cell proliferation determined by XTT assay. *, *P* < 0.01; * *, *P* < 0.001. **c** Cell migration activity determined with wound healing assay. *, *P* < 0.01; * *, *P* < 0.001. **d** Cell invasion activity determined with Matrigel invasion assay. *, *P* < 0.001. **e** Western blot analysis of apoptotic markers (cleaved PARP and PARP) in CDDP-R-BOY and CDDP-R-T24. The full-length blots/gels are presented in Supplementary Figure 8.

First, we used qRT-PCR to confirm that expression of *miRNA-486-5p* was downregulated in CDDP-R BC cells compared with parental cells (Fig. 3a). We also performed gain-of-function studies of parental cell lines (BOY and T24) and CDDP-R cell lines (CDDP-R-BOY and CDDP-R-T24) transfected with *miRNA-486-5p* (Supplementary Figure 3a) to investigate the functional roles of *miRNA-486-5p*. Cell proliferation of both parental and CDDP-R BC cells transfected with *miRNA-486-5p* was significantly inhibited in the XTT assay in comparison with mock or microRNA-control transfected cells (Fig. 3b). Moreover, cell migration activity in wound healing assays and cell invasion in Matrigel invasion assays showed significant inhibition in the *miRNA-486-5p* transfectants compared to their counterparts (Fig. 3c, d, Supplementary Figure 3b, c). Because cisplatin induces apoptosis in cancer cells [5], we used Western blots to assess apoptosis. The expression level of cleaved PARP increased in *miRNA-486-5p* transfectants (Fig. 3e). Thus, *miRNA-486-5p* induced apoptosis in both parental and CDDP-R BC cells and provided anti-tumor effects.

### Transfection of *miRNA-486-5p* increased the sensitivity of CDDP-R-BC cell lines to cisplatin

**Fig. 4:**
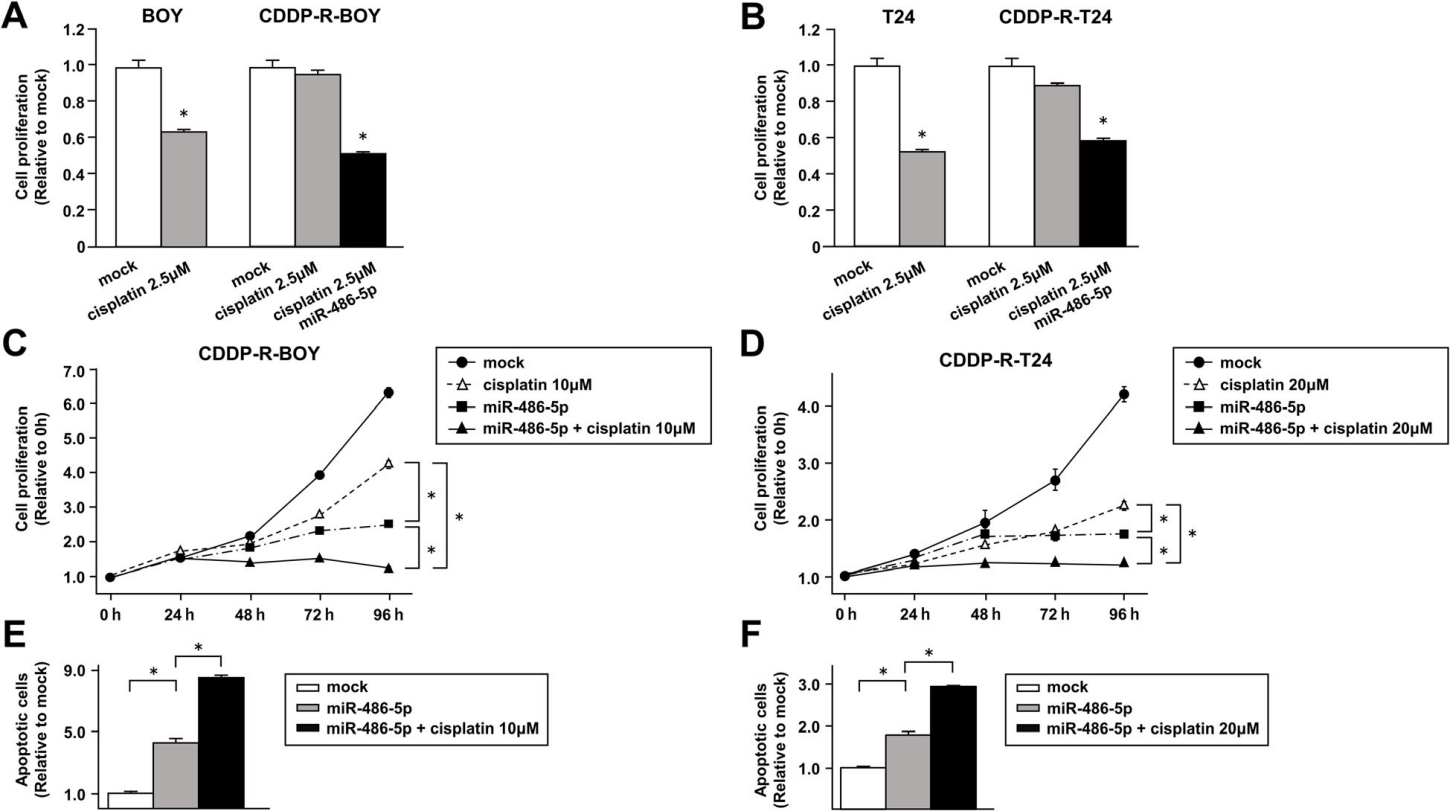
Transfection of *miRNA-486-5p* improves sensitivity to CDDP in CDDP-R-BC cell lines. The IC50 concentration of cisplatin was given to parental and cisplatin-resistant cells. Transfection of 10 nM *miRNA-486-5p* increased the cisplatin sensitivity in (**a**) CDDP-R-BOY and (**b**) CDDP-R-T24 cells as determined by XTT assay. *, *P* < 0.001. *miRNA-486-5p* transfection and cisplatin administration (IC50 dose) to (**c**) CDDP-R-BOY and (**d**) CDDP-R-T24 cells clearly had an additive effect and significantly suppressed cell proliferation. *, *P* < 0.001. **e, f** Apoptosis assays were carried out using flow cytometric analysis of CDDP-R-BC cells. *, *P* < 0.001

As shown in Fig. 1c, cell viability was not suppressed in CDDP-R-BOY by treatment with 2.5 μM cisplatin, which is the IC50 concentration for BOY. However, by simultaneous *miRNA-486-5p* transfection, cell proliferation decreased to the level observed in parental BOY (Fig. 4a) at 2.5 μM cisplatin. Similar results were found with T24 (Fig. 4b). Next, we examined cell proliferation following cisplatin treatment and *miRNA-486-5p* transfection. We administered 10 μM cisplatin, which is the IC50 for CDDP-R-BOY. The combination of *miRNA-486-5p* transfection and cisplatin administration clearly had additive effects and significantly suppressed cell proliferation (Fig. 4c). We also administered 20 μM cisplatin to CDDP-R-T24 and obtained similar results (Fig. 4d). The combination of *miRNA-486-5p* transfection and cisplatin induced more apoptotic cells in flow cytometric analyses (Fig. 4e, f Supplementary Figure 4a). Thus, cell proliferation was suppressed and apoptosis was enhanced. These results suggested that *miRNA-486-5p* functioned as a tumor suppressor in CDDP-R cells and increased their sensitivity to cisplatin.

### Identification of *EHHADH* mRNA as a target regulated by *miR-486-5p* in CDDP-R-BC cell lines

**Fig. 5:**
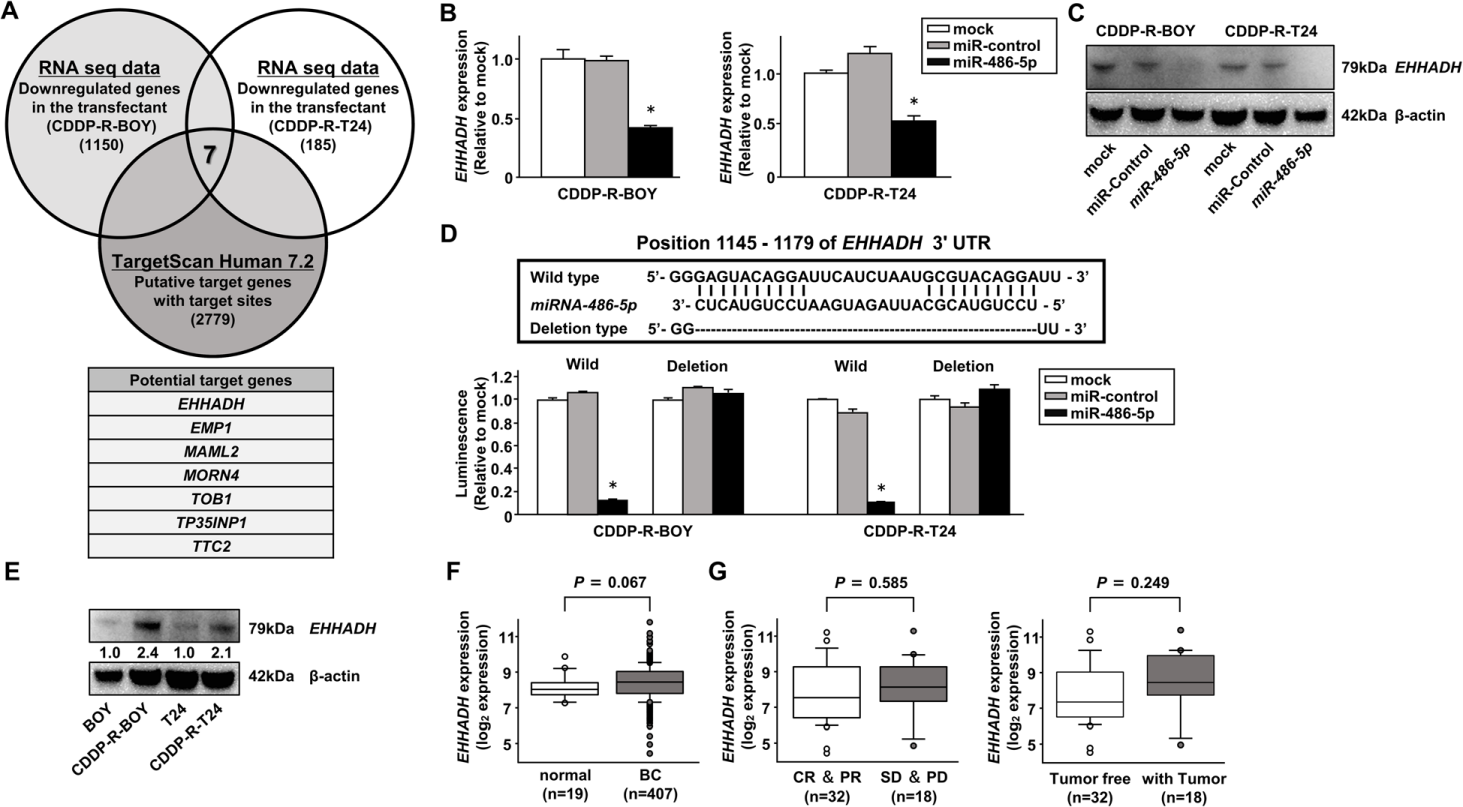
Identification of *EHHADH* mRNA as a target regulated by *miR-486-5p* in CDDP-R-BC cell lines. **a** A Venn diagram of mRNA sequences and in silico analyses indicated that 7 putative candidate target genes of *miRNA-486-5p* were key molecules in cisplatin-resistant BCs. **b** The *EHHADH* mRNA expression levels determined by qRT-PCR and (**c**) its protein levels determined by Western blot in *miR-486-5p* transfectants compared with mock or miR-control transfectants. *, *P* < 0.001. The full-length blots/gels are presented in Supplementary Figure 8. **d** Dual-luciferase reporter assays using vectors encoding putative miRNA target sites for WT or deleted regions. Luminescence intensity was significantly reduced by co-transfection with *miR-486-5p* and the vector carrying the WT 3′-UTR. *, *P* < 0.001. **e** The protein levels of EHHADH in CDDP-R BC and parental BC cells. The full-length blots/gels are presented in Supplementary Figure 8. **f** *EHHADH* mRNA expression in the BLCA cohort in TCGA, *EHHADH* expression in BLCA samples compared with those in normal samples. **g** *EHHADH* expression in cisplatin-sensitive (CR and PR) and cisplatin-resistant (SD and PD) groups treated with cisplatin-based chemotherapy. *EHHADH* expression in patients with or without tumor

Next, we sought further insights into the molecular mechanisms regulated by tumor suppressive *miRNA-486-5p*. Thus, we used a combination of in silico analyses and RNA sequencing analyses to search for genes in CDDP-R BC cells that were targeted by *miRNA-486-5p*. TargetScan database Release 7.1 (http://www.targetscan.org) identified 2779 possible mRNAs as candidate targets of *miRNA-486-5p*. Next, we narrowed the number of genes based upon the expression profiles of mRNAs of CDDP-R BC cell lines before and after transfection with *miRNA-486-5p*. Finally, we selected 7 candidate target genes (*EHHADH, EMP1, MAML2, MORN4, TOB1, TP35INP1, TTC2*) (Fig. 5a). Among them, only *EHHADH* gene expression was downregulated at both the mRNA and the protein expression level in *miRNA-486-5p*-transfected cells (Fig. 5b, c, Supplementary Figure 5b).

To determine whether *EHHADH* was directly regulated by *miRNA-486-5p*, we performed dual-luciferase reporter assays in CDDP-R BC cell lines. The TargetScan database predicted that there were 2 binding sites for *miRNA-486-5p*. We used vectors encoding the partial WT sequence of the 3′-UTR of *EHHADA*, including the predicted *miRNA-486-5p* target sites. The luminescence intensity was significantly reduced by co-transfection with *miRNA-486-5p* and the vector carrying the WT 3′-UTR, whereas it was not reduced by transfection with the deletion vector from which the binding site had been removed (Fig. 5d). These data suggested that *miRNA-486-5p* was directly bound to each specific position in the 3′-UTR of *EHHADH* mRNA.

### Expression levels of *EHHADH* correlated with cisplatin resistance in BC in TCGA cohorts and in vitro

We examined the correlation of *EHHADH* expression levels with cisplatin sensitivities and clinical categories. We confirmed that the protein level of EHHADH was increased in CDDP-R BC cell lines compared with parental cell lines (Fig. 5e). Among the BLCA cohort in TCGA, we found that gene expression of *EHHADH* was somewhat higher in bladder cancer specimens compared with adjacent noncancerous tissues (*P* = 0.067; Fig. 5f). However, there were no significant differences in the expression levels of *EHHADH* among clinicopathological parameters (Supplementary Figure 5c). Thus, we examined patients who were treated with cisplatin-based chemotherapy. The cohort was divided into two groups: (1) a cohort that included CR (Complete Response) and PR (Partial Response) patients that showed tumor suppression due to cisplatin-based chemotherapy and (2) a cohort that included SD (Stable Disease) and PD (Progressive Disease) patients who did not show tumor suppression due to cisplatin resistance [26]. Even though we could not find a significant difference between in the CR and PR group and in the SD and PD group, the expression level of *EHHADH* in the SD and PD group tended to be a little higher than that in the CR and PR group (Fig. 5g), which was different from the data showing no difference in the expression levels of EHHADH among clinicopathological parameters in Supplementary Figure 5C. When we separated the cohort into a tumor-free group and a residual tumor group, there was also a tendency that the expression of *EHHADH* was higher in the tumor-free group than in the tumor-free group.

### Effects of *EHHADH* knockdown on cell proliferation, migration, invasion, and apoptosis in CDDP-R BC cells

**Fig. 6:**
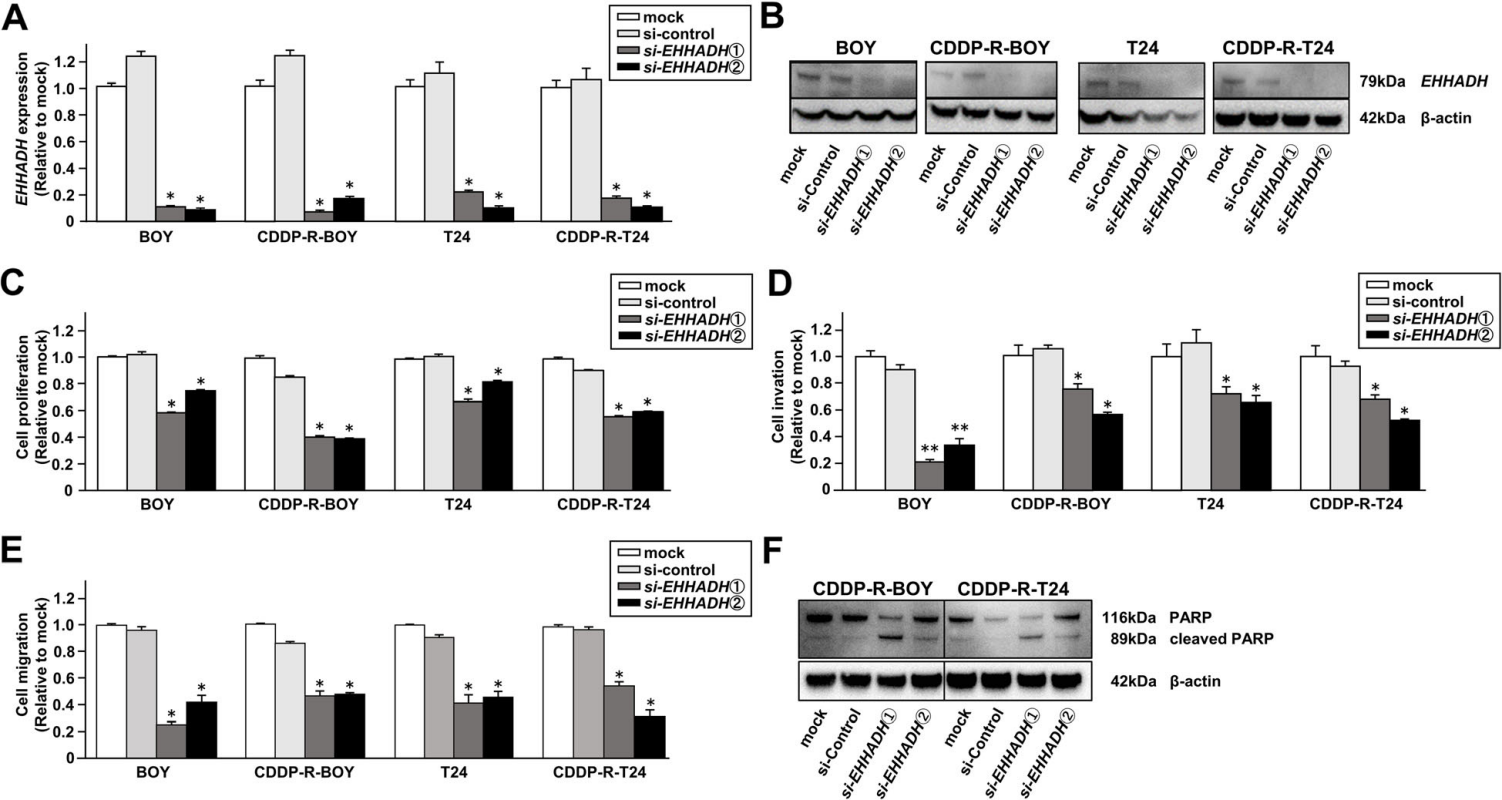
Effects of *EHHADH* knockdown on cell proliferation, migration, invasion, and apoptosis in CDDP-R BC cells. **a** *EHHADH mRNA* expression [measured by RT-qPCR] and (**b**) EHHADH protein levels [measured by Western blot analyses] were assessed to validate the knockdown efficiency of *si-EHHADH*. *, *P* < 0.001. **c** Cell proliferation determined by XTT assay. *, *P* < 0.001. The full-length blots/gels are presented in Supplementary Figure 8. **d** Cell invasion activity determined with the Matrigel invasion assay. *, *P* < 0.001. **e** Cell migration activity determined with wound healing assays. *, *P* < 0.001; **, *P* < 0.01. All experiments were performed in quadruplicate and *si-EHHADH* transfectants were compared with mock or si-control transfectants. **f** Western blot analysis of apoptotic markers (cleaved PARP and PARP) in CDDP-R-BOY and CDDP-R-T24. The full-length blots/gels are presented in Supplementary Figure 8.

We carried out loss-of-function assays by using *si-EHHADH* transfections to investigate the functional role of *EHHADH* in CDDP-R-BC cells. We employed 2 different *si-EHHADH* molecules that effectively downregulated *EHHADH* mRNA and protein expression in both cell lines (Fig. 6a, b). Transfections of si-EHHADH did not regulate *miRNA-486-5p* expression (Supplementary Figure 6a). XTT assays demonstrated that cell proliferation was significantly inhibited in both parental and CDDP-R cells transfected with *si-EHHADH* in comparison with mock or si-control transfected cells. Moreover, Matrigel invasion assays and wound healing assays showed that cell invasion and migration activities were significantly inhibited in these *si-EHHADH* transfectants compared to their counterparts (Fig. 6c, d, e, Supplementary Figure 6b, c). The apoptotic cell numbers were significantly greater in *si-EHHADH* transfectants than in their counterparts. Western blots showed that cleaved PARP expression was markedly increased in *si-EHHADH* transfectants.

### Knockdown of *EHHADH* improves sensitivity to CDDP in CDDP-R-BC cell lines

**Fig. 7:**
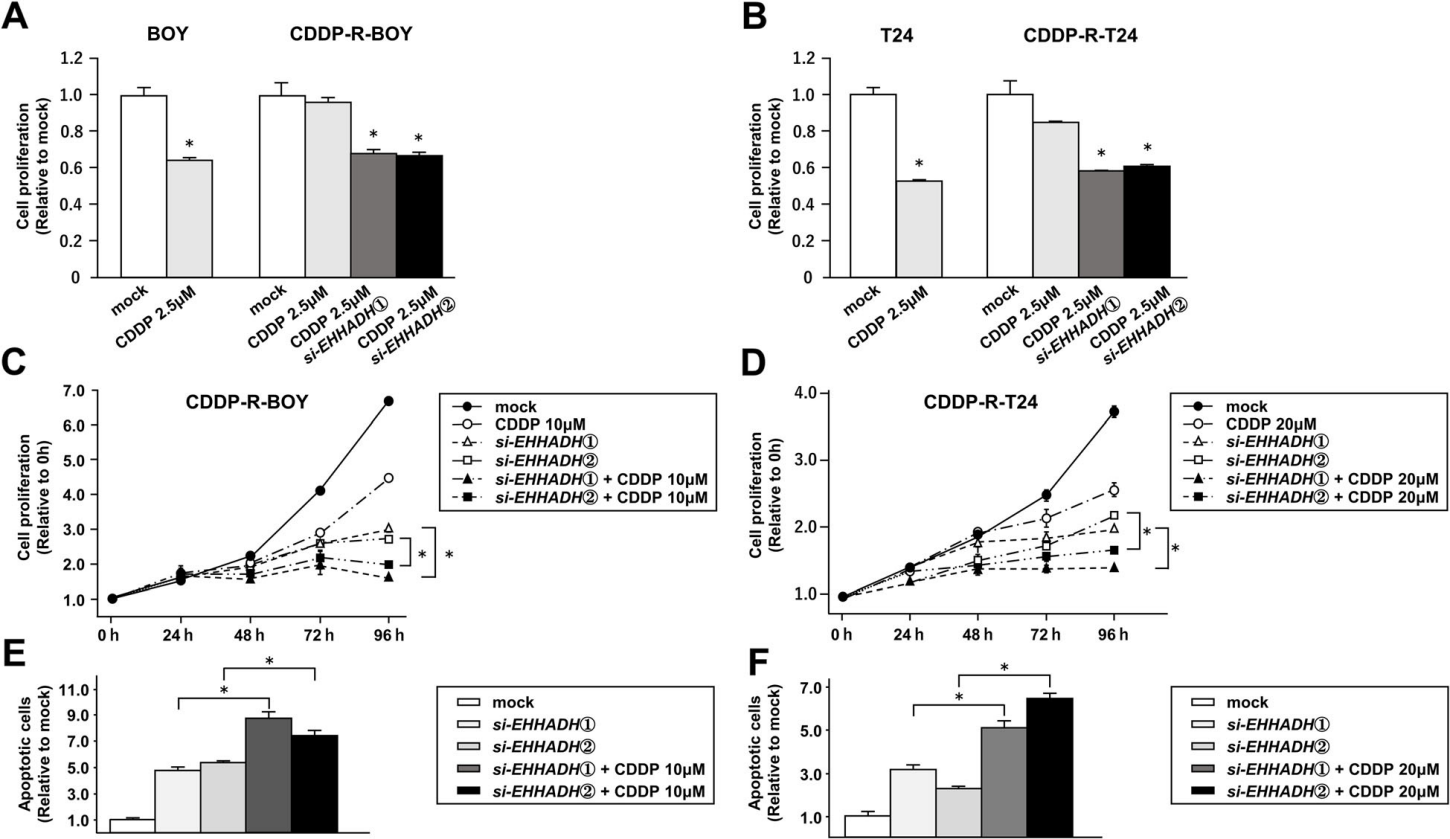
Transfection of *si-EHHADH* increased sensitivity to CDDP in CDDP-R-BC cell lines. The IC50 concentration of cisplatin was given to parental BC cells. Transfection of 10 nM *si-EHHADH* increased the cisplatin sensitivity in (**a**) CDDP-R-BOY and (**b**) CDDP-R-T24 cells as determined by XTT assay. *, *P* < 0.001. The combination of si-*EHHADH* transfection and cisplatin administration to (**c**) CDDP-R-BOY and (**d**) CDDP-R-T24 cells clearly had an additive effect and significantly suppressed cell proliferation. *, *P* < 0.001. **e**, **f** Apoptosis assays were carried out using flow cytometric analysis of CDDP-R-BC cells. *, *P* < 0.001

We also investigated whether knockdown of *EHHADH* improved the sensitivity to cisplatin in CDDP-R-BC cells. The cell proliferation of CDDP-R-BOY was not suppressed at 2 μM cisplatin, however a combination of *si-EHHADH*-transfection and cisplatin clearly had an additive effect and significantly suppressed cell proliferation at 2 μM cisplatin (Fig. 7a). Similar results were observed in CDDP-R-T24 with 10 μM of cisplatin (Fig. 7b). Next, we observed the proliferative activity after combining cisplatin and *si-EHHADH* transfection in a time series and found that the combination treatment clearly inhibited cell proliferation compared to the individual treatments (Fig. 7c, d). Further, the number of apoptotic cells (apoptotic and early apoptotic cells) was significantly higher in the combination treatment (Fig. 7e, f, Supplementary Figure 7a). These results suggested that *EHHADH* was involved in cisplatin resistance in BC and that its inhibition might improve the cells’ sensitivity to cisplatin.

## Discussion

*EHHADH* is 1 of the 4 enzymes of the peroxisomal beta-oxidation pathway [27]. Beta-oxidation of fatty acids occurs in both mitochondria and peroxisomes. The preponderance of activity is found in mitochondria where fatty acid molecules are disassembled to acetyl-CoA, which is oxidized to CO_2_ in the citric acid cycle to produce energy [27, 28]. Peroxisomal beta-oxidation is involved in the decomposition of very long chain fatty acids, bile acid synthesis and myelin sheath lipid synthesis [29, 30]. Recently, peroxisomes have gained attention in human health with potential impact on a large number of diseases such as neurodegeneration, age-related disorders, and cancer [31]. The peroxisome proliferator-activated receptors are involved in the peroxisomal beta-oxidation pathway and are believed to affect cardiovascular disorders, diabetes, neurological and psychiatric disorders and malignancies [32].

In cancer cells, abnormal expression of microRNAs can disturb normally operating RNA networks and disrupt physiologic processes [11]. microRNAs are important regulators that control cancer progression pathways, including cell proliferation, differentiation, development, apoptosis and drug resistance [33, 34]. Our past studies demonstrated that specific miRNAs are abnormally expressed and impact cancer progression through their targeting of several oncogenic genes and pathways [35–37]. Other researchers have reported that some microRNAs are involved in cisplatin resistance in BC. Liu et al. showed that miRNA-214 could reduce cisplatin resistance by targeting netrin-1 [38]. Li et al. demonstrated that decreased expression of miRNA-218 could contribute to resistance to cisplatin by suppressing glucose metabolism [39]. Herein, we focused on *miRNA-486-5p*, a transcript that was downregulated in CDDP-R BC cell lines (CDDP-R-BOY, CDDP-R-T24) compared with parental BC cell lines (BOY, T24). The molecular mechanism by which *miRNA-486-5p* is downregulated in BC remains unclear. However, *miRNA-486-5p* is located on chromosome 8p11, and loss of material from chromosome arm 8p was a frequent cytogenetic alteration in uroepithelial carcinoma [40]. It was reported that *miRNA-486-5p* was a tumor suppressor miRNA in many cancer types such as non-small cell lung, breast, colon and hepatocellular carcinoma [41–44]. As a tumor suppressor gene, *miRNA-486-5p* overexpression inhibited cell proliferation, migration and invasion, and induced apoptosis in BC and CDDP-R BC cell lines. Also, *miRNA-486-5p* was reportedly involved in sensitivity to cisplatin in non-small cell lung cancer. Xiaoyan et al. reported that *miRNA-486-5p* inhibits *EMT* by targeting *TWF1* and improves sensitivity to cisplatin [45]. Similarly, we revealed that *miRNA-486-5p* overcomes cisplatin resistance and the additive effect on cell growth suppression. Recently, Salimian et al. showed that *miRNA-486-5p* had anti-tumor effects and improved CDDP sensitivity through induction of apoptosis in muscle-invasive BC [46]. Therefore, Our finding adds a new perspective to their reports, we demonstrated that *EHHADH* was a directly targeted by *miR-486-5p.* This finding had not been reported previously. According to TargetScan database Release 7.1, it is possible that among the 28 microRNAs that are downregulated in cisplatin-resistant BC cell lines (CDDP-R-BOY, CDDP-R-T24), 5 microRNAs (*miR-486-5p*, *miR-6768-5p, miR-548ar-3p, miR-6816-3p, miR-4731-3p*) regulate the expression of *EHHADH*. Even though we focused on *miR-486-5p* in this study, it is possible that the other microRNAs are involved in cisplatin resistance by regulating *EHHADH*. Further study is necessary to elucidate the mechanism by which EHHADH contributes to cisplatin resistance through regulation by microRNA in BC.

In this study, we confirmed that the level of EHHADH protein was elevated in CDDP-R BC cell lines compared with parental cell lines, and that loss of *EHHADH* gene function significantly inhibited cancer cell proliferation, migration and invasion and increased the cells’ sensitivity to cisplatin. However, we could not find a significant correlation between *EHHADH* expression levels and cisplatin-based chemotherapy response in TCGA database. This could be due to the limited number of samples in TCGA database. Also, mRNA expression does not always match protein expression. Furthermore, when cisplatin-based chemotherapy is used, cisplatin is combined with other anticancer agents. Because our analysis showed a tendency for upregulation of *EHHADH* expression in patients with cisplatin resistance in spite of the small sample size and bias, the results suggest that *EHHADH* could be a new molecular target and marker for progressive BC.

The mechanisms by which cells gain resistance to cisplatin are very complex. Thus, Galluzzi et al. classified the mechanisms of resistance into 4 categories [47]. The first category is pre-target resistance in which the binding of cisplatin to DNA is reduced, perhaps because of lowered cisplatin uptake into cells. The second category is on-target resistance due to inadequate direct binding between DNA and cisplatin. The third category is post-target resistance in which cisplatin-mediated DNA damage is ineffective. The last category is off-target resistance in which no signaling pathway is triggered by cisplatin. For example, the upregulation of excision repair cross complementing 1 (*ERCC1*), a DNA repair gene [48], could repair DNA damage caused by addition of cisplatin [49]. This cisplatin resistance mechanism is classified as on-target resistance [47]. In another example, TP53 mutant patients are often cisplatin-resistant in ovarian cancer [50]. Because *TP53* is a tumor suppressor gene that mainly induces apoptosis [51], patients with a faulty *TP53* gene cannot proceed to cellular apoptosis (post-target resistance) [47]. Regarding *EHHADH,* it appears to contribute to pre-target resistance for the following reasons. First, Evelien et al. reported that increased fatty acid synthesis reduces intracellular unsaturated fatty acid production. Unsaturated fatty acids are a source of reactive free radicals, and reduction of reactive free radicals is involved in cisplatin resistance [52]. Second, Chiranjeevi et al. demonstrated that fatty acid synthesis may permit plasma membrane remodeling by varying the fatty acid and lipid composition [53, 54]. This variation could lead to altered drug uptake and intracellular drug concentration, affecting drug resistance. Because it remains unclear how the functions of *EHHADH* cause cisplatin resistance, further studies are necessary to elucidate the associations between the peroxisomal beta-oxidation pathway in which *EHHADH* is involved and cisplatin resistance.

## Conclusions

We identified *EHHADH* as a novel target *of miRNA-486-5p* in cisplatin-resistant BCs. The expression of *EHHADH* was decreased in cisplatin-resistant BC cell lines. To the best of our knowledge, this is the first report demonstrating that *EHHADH* is involved in cisplatin resistance. The discovery of molecular targets mediated by tumour-suppressive microRNAs may lead to a better understanding of the mechanisms of cisplatin resistance in BC and the development of new therapeutic strategies to treat progressive BC.

## Supplementary Information

**Additional file 1 **: **Supplementary Figure 1. (a)** Cell proliferation determined by XTT assay compared with parental BC cell lines and CDDP-R BC cell lines. *, *P* < 0.001. **(b)** Heatmap of miRNA-seq comparing parental and CDDP-R cell lines (BOY vs CDDP-R-BOY, T24 vs CDDP-R-T24).

**Additional file 2 **: **Supplementary Figure 2. (a)** Among the BLCA cohort of TCGA, expression levels of candidate miRNAs (*miRNA-624-3p, miRNA-424-5p, miRNA-545-5p, miR-628-3p*). We determined correlations among expression levels and pathological T categories and clinical stages. **(b)** Kaplan-Meier analysis using TCGA dataset revealed that the high *miRNA-545-5p* expression group did not have significantly lower OS than the low *miRNA-486-5p* expression group (*P* = 0.162).

**Additional file 3 **: **Supplementary Figure 3. (a)** Expression levels of *miRNA-486-5p* quantified in *miR-486-5p* transfectants compared with mock or miR-control transfectants by qRT-PCR. **(b)** Pictures of cell invasion assays. **(c)** Pictures of cell migration assays.

**Additional file 4 **: **Supplementary Figure 4. (a)** Apoptosis assays indicated that the number of apoptotic cells was significantly greater in the combination of miRNA-486-5p-transfection and cisplatin than single treatment in flow cytometry, *P < 0.0001.

**Additional file 5 **: **Supplementary Figure 5. (a)** Heatmap of mRNA-seq comparing mock with *miRNA-486-5p* transfection in CDDP-R cell lines (CDDP-R-BOY, CDDP-R-T24). **(b)** Expression levels of *miRNA-486-5p* quantified in *miR-486-5p* transfectants compared with mock or miR-control transfectants by qRT-PCR. **(c)** Among the BLCA cohort of TCGA, there were no significant differences in expression levels of *EHHADH* in pathological categories or clinical stages.

**Additional file 6 **: **Supplementary Figure 6. (a)** Expression levels of *miRNA-486-5p* quantified in *si-EHHADH* transfectants compared with mock or si-control transfectants determined by qRT-PCR. **(b, c)** Pictures of cell invasion assays and cell migration assays in si-*EHHADH* transfectants compared with mock or si-control transfectants.

**Additional file 7 **: **Supplementary Figure 7. (a)** Apoptosis assays indicated that the number of apoptotic cells was significantly greater in the combination of si-EHHADH-transfection and cisplatin than single treatment in flow cytometry, *P < 0.0001. **(b)** Correlations of the expression of miRNA-545-5p and EHHADH in bladder cancer samples in TCGA database.

**Additional file 8.**

## Data Availability

The datasets used and analyzed during the current study are available from the corresponding author on reasonable request.

## Abbreviations

*EHHADH*: Enoyl-CoA, hydratase/3-hydroxyacyl CoA dehydrogenase
BC: Bladder cancer
microRNA: Microribonucleic acid
CDDP-R: Cisplatin-resistant
TCGA: The Cancer Genome Atlas
NMIBC: Non-muscle-invasive bladder cancer
MIBC: Muscle-invasive bladder cancer
OS: Overall survival rate
*CTR1*: Copper transporter1
EMT: Epithelial mesenchymal transition
*PTEN*: Phosphatase and Tensin Homolog deleted on Chromosome 10
*AKT*: RAC-alpha serine/threonine-protein kinase
*BCL2*: B-cell lymphoma 2
IC50: Half maximal inhibitory concentration
BLCA: Bladder urothelial carcinoma
UTR: Untranslated Region
ERCC1: Excision repair cross complementing 1
*TP53*: Tumor protein p53
