# Supplementary figures and images for: EHHADH contributes to cisplatin resistance through regulation by tumor-suppressive microRNAs in bladder cancer
Source: BMC Cancer. 2021 Jan 11;21:48. doi: 10.1186/s12885-020-07717-0 (PMC7798329; doi:10.1186/s12885-020-07717-0)

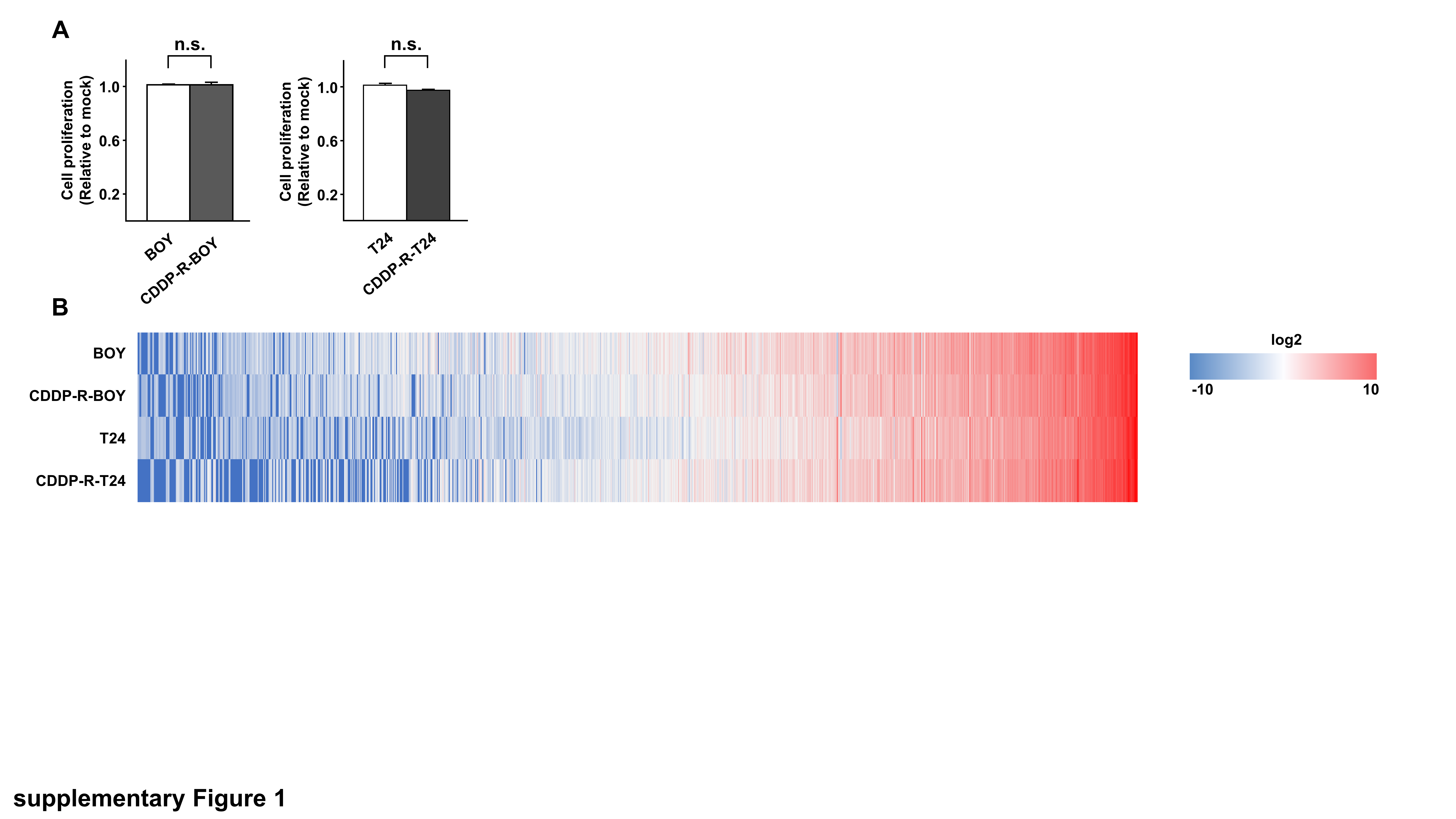

Supplement: Supplementary file 1 — Additional file 1 : Supplementary Figure 1. (a) Cell proliferation determined by XTT assay compared with parental BC cell lines and CDDP-R BC cell lines. *, P < 0.001. (b) Heatmap of miRNA-seq comparing parental and CDDP-R cell lines (BOY vs CDDP-R-BOY, T24 vs CDDP-R-T24). [file 12885_2020_7717_MOESM1_ESM.tif]

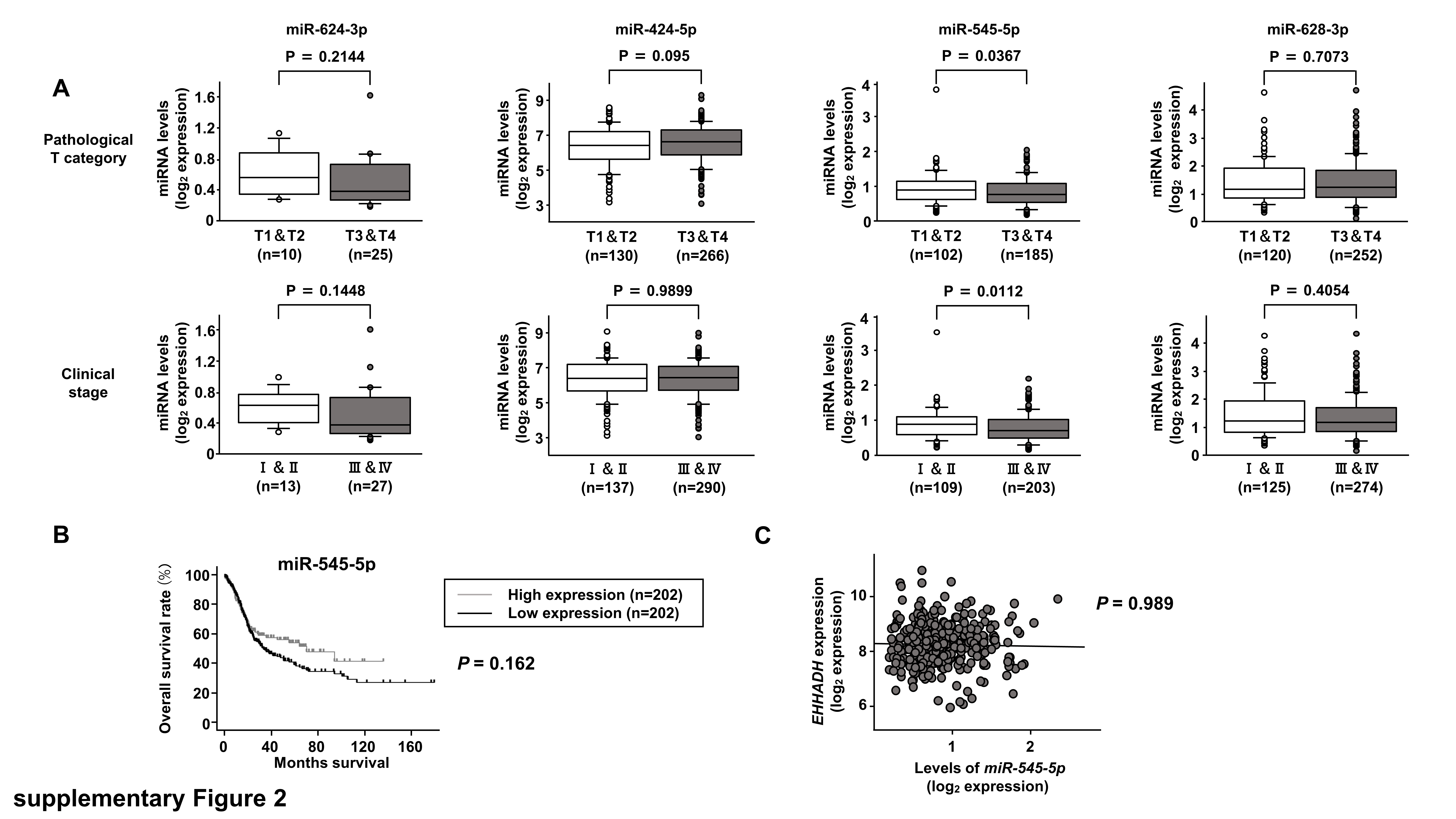

Supplement: Supplementary file 2 — Additional file 2 : Supplementary Figure 2. (a) Among the BLCA cohort of TCGA, expression levels of candidate miRNAs (miRNA-624-3p, miRNA-424-5p, miRNA-545-5p, miR-628-3p). We determined correlations among expression levels and pathological T categories and clinical stages. (b) Kaplan-Meier analysis using TCGA dataset revealed that the high miRNA-545-5p expression group did not have significantly lower OS than the low miRNA-486-5p expression group (P = 0.162). [file 12885_2020_7717_MOESM2_ESM.tif]

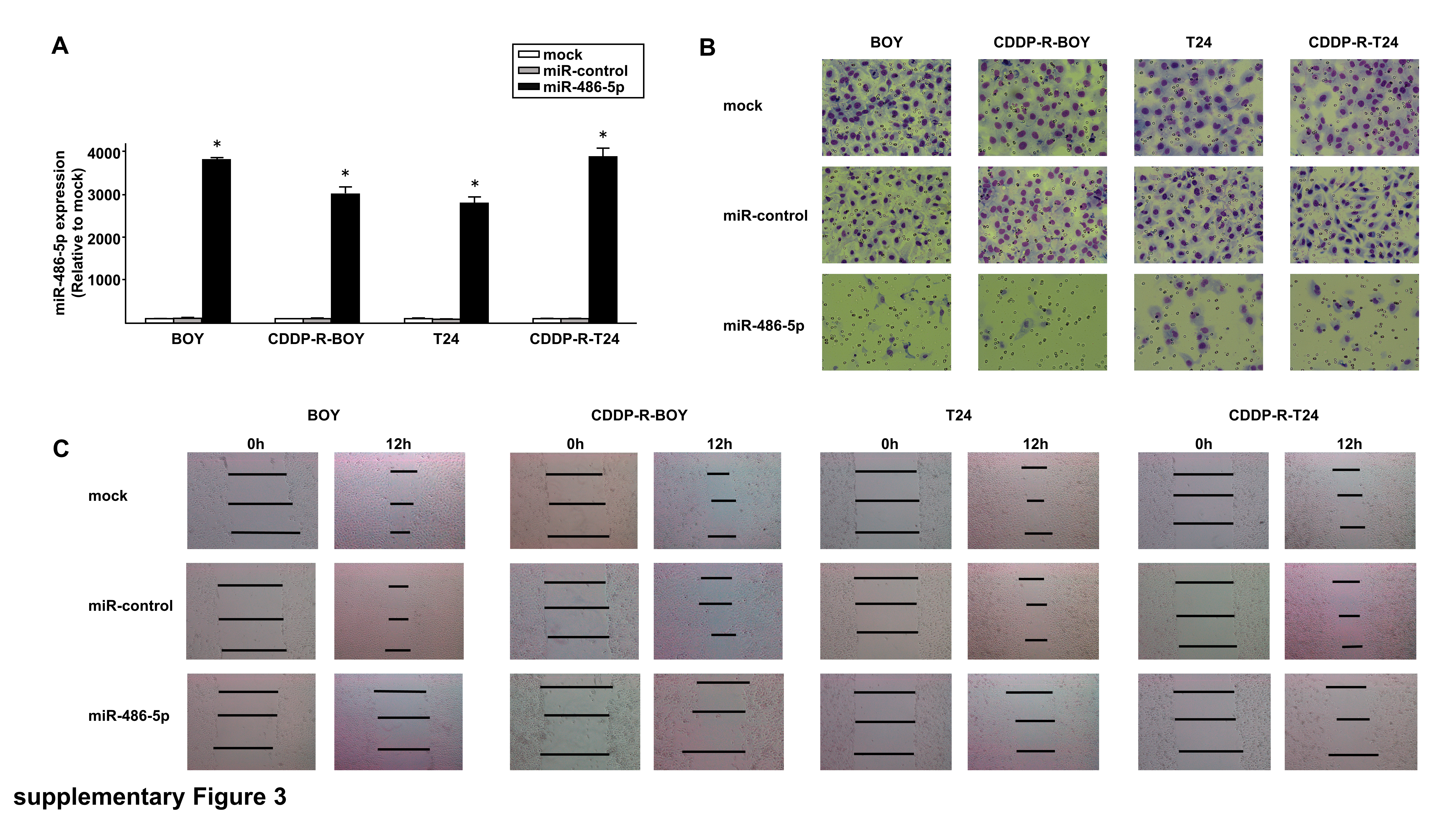

Supplement: Supplementary file 3 — Additional file 3 : Supplementary Figure 3. (a) Expression levels of miRNA-486-5p quantified in miR-486-5p transfectants compared with mock or miR-control transfectants by qRT-PCR. (b) Pictures of cell invasion assays. (c) Pictures of cell migration assays. [file 12885_2020_7717_MOESM3_ESM.tif]

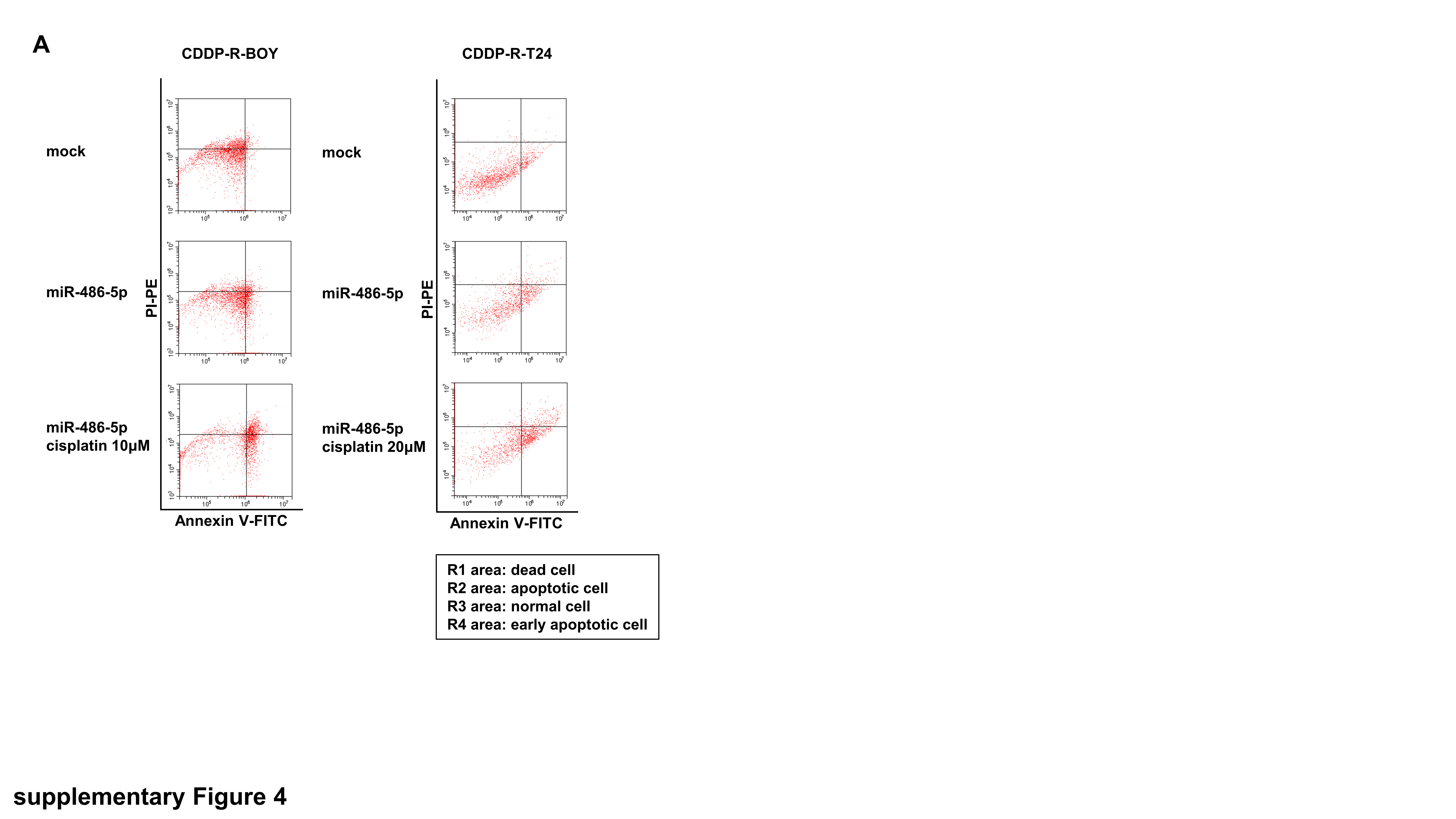

Supplement: Supplementary file 4 — Additional file 4 : Supplementary Figure 4. (a) Apoptosis assays indicated that the number of apoptotic cells was significantly greater in the combination of miRNA-486-5p-transfection and cisplatin than single treatment in flow cytometry, *P < 0.0001. [file 12885_2020_7717_MOESM4_ESM.tif]

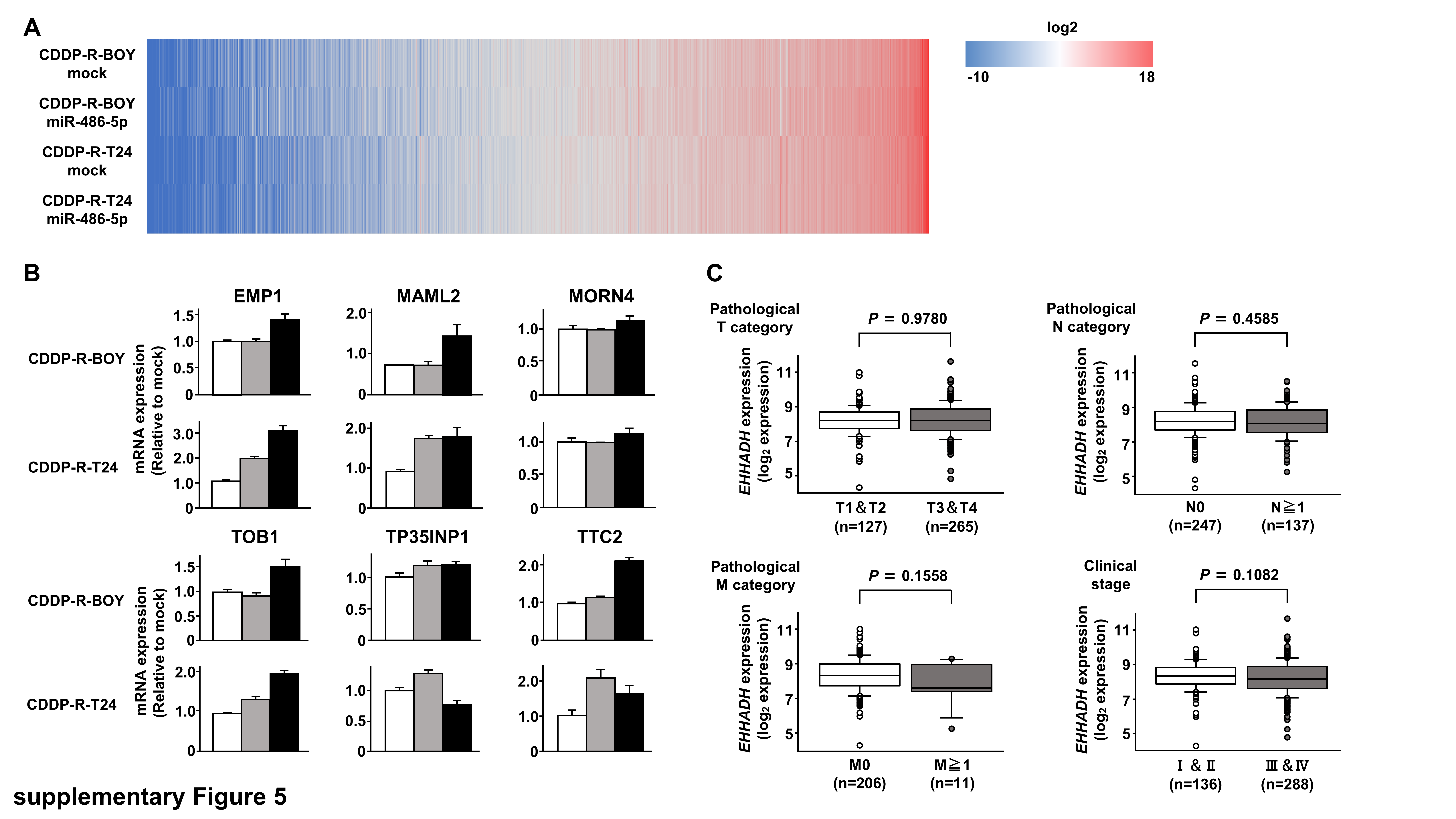

Supplement: Supplementary file 5 — Additional file 5 : Supplementary Figure 5. (a) Heatmap of mRNA-seq comparing mock with miRNA-486-5p transfection in CDDP-R cell lines (CDDP-R-BOY, CDDP-R-T24). (b) Expression levels of miRNA-486-5p quantified in miR-486-5p transfectants compared with mock or miR-control transfectants by qRT-PCR. (c) Among the BLCA cohort of TCGA, there were no significant differences in expression levels of EHHADH in pathological categories or clinical stages. [file 12885_2020_7717_MOESM5_ESM.tif]

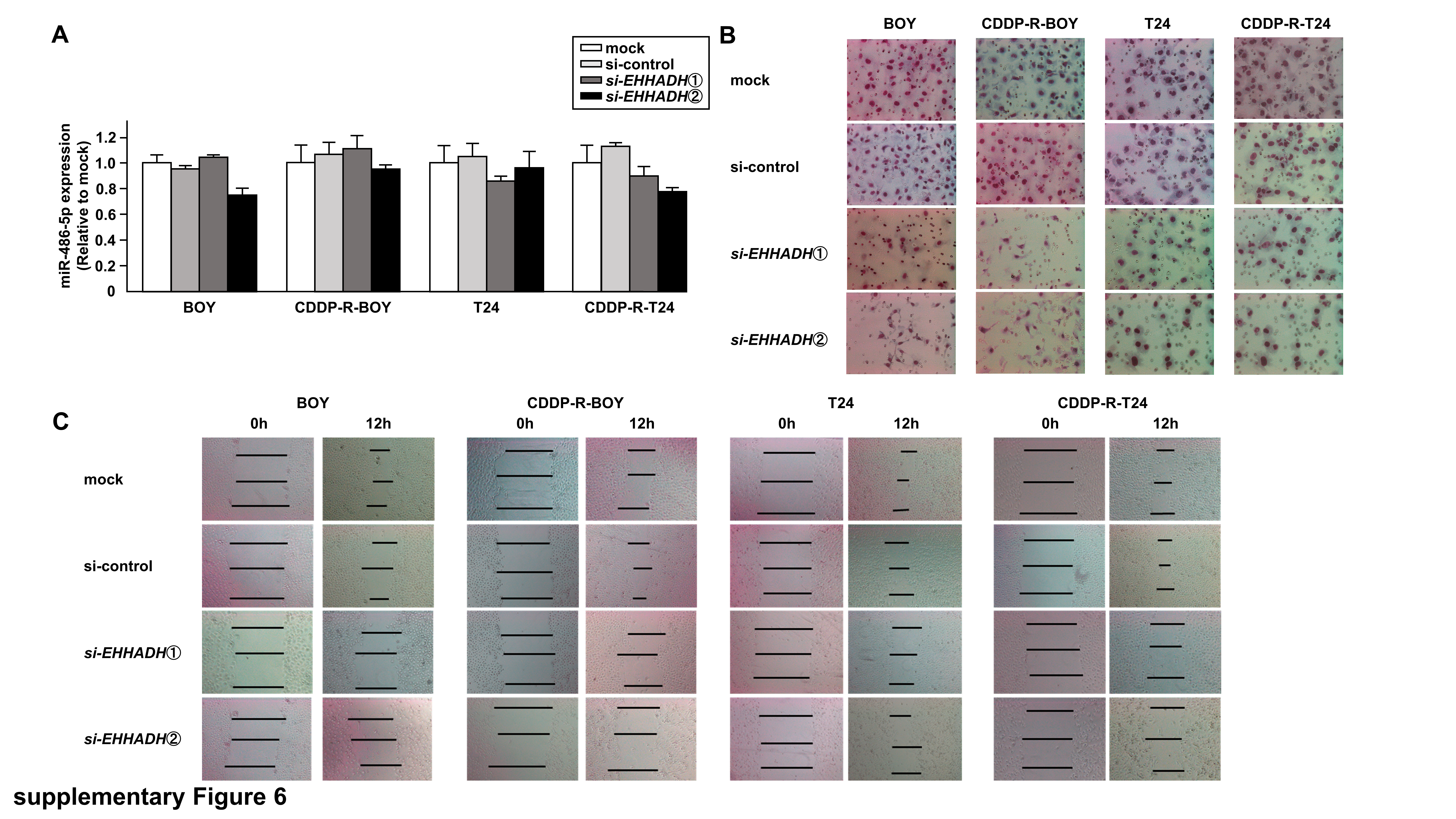

Supplement: Supplementary file 6 — Additional file 6 : Supplementary Figure 6. (a) Expression levels of miRNA-486-5p quantified in si-EHHADH transfectants compared with mock or si-control transfectants determined by qRT-PCR. (b, c) Pictures of cell invasion assays and cell migration assays in si-EHHADH transfectants compared with mock or si-control transfectants. [file 12885_2020_7717_MOESM6_ESM.tif]

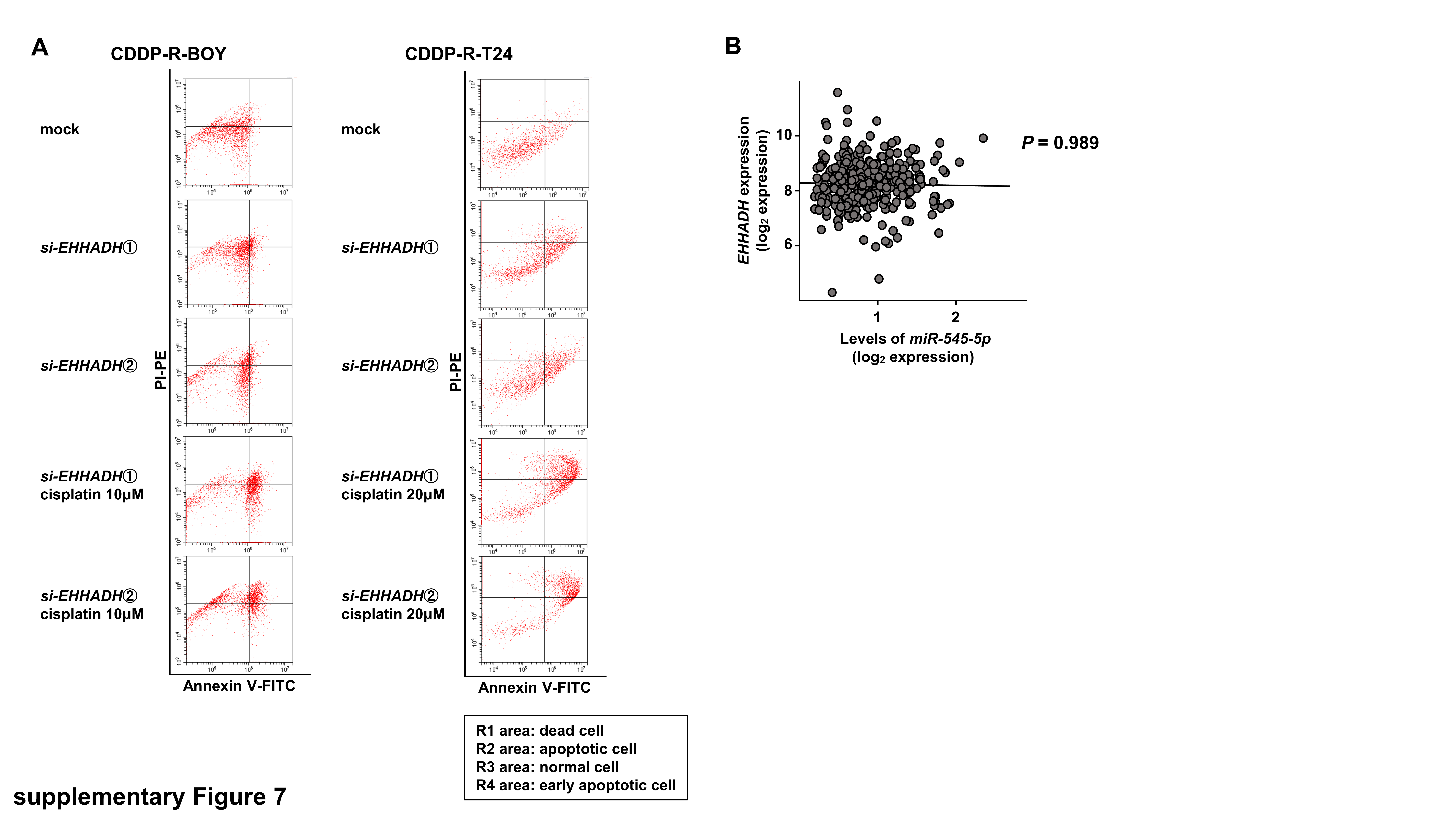

Supplement: Supplementary file 7 — Additional file 7 : Supplementary Figure 7. (a) Apoptosis assays indicated that the number of apoptotic cells was significantly greater in the combination of si-EHHADH-transfection and cisplatin than single treatment in flow cytometry, *P < 0.0001. (b) Correlations of the expression of miRNA-545-5p and EHHADH in bladder cancer samples in TCGA database. [file 12885_2020_7717_MOESM7_ESM.tif]
