# Supplementary material for: EHHADH contributes to cisplatin resistance through regulation by tumor-suppressive microRNAs in bladder cancer
Source: BMC Cancer. 2021 Jan 11;21:48. doi: 10.1186/s12885-020-07717-0 (PMC7798329; doi:10.1186/s12885-020-07717-0)
Supplement: Supplementary file 8 — Additional file 8. [file 12885_2020_7717_MOESM8_ESM.pptx]

## Slide 1
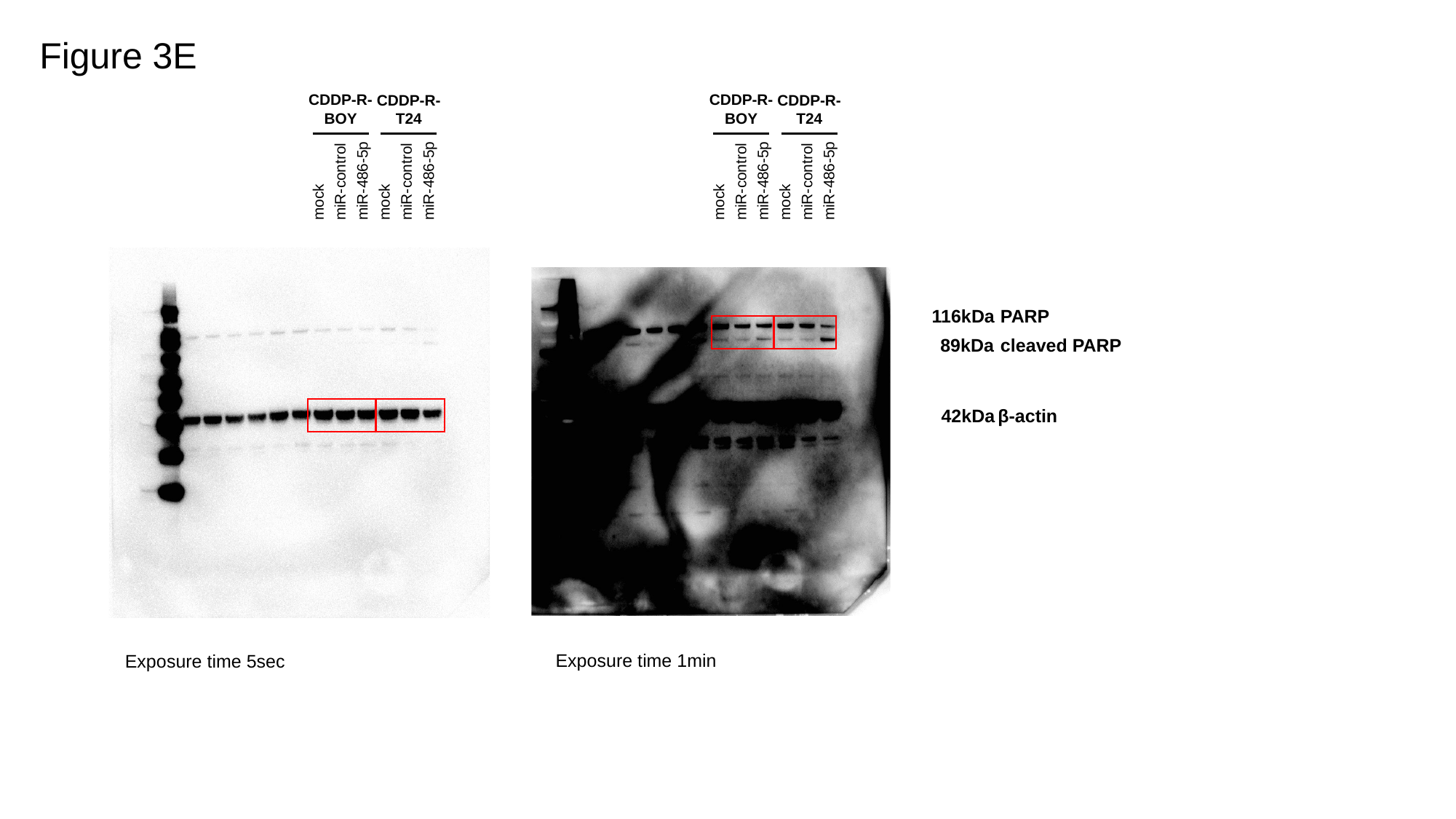

Figure 3E
CDDP-R-
BOY
CDDP-R-
BOY
CDDP-R-
T24
CDDP-R-
T24
mock
miR-control
miR-486-5p
mock
miR-control
miR-486-5p
mock
miR-control
miR-486-5p
mock
miR-control
miR-486-5p
116kDa
PARP
89kDa
cleaved PARP
42kDa
β-actin
Exposure time 1min
Exposure time 5sec

## Slide 2
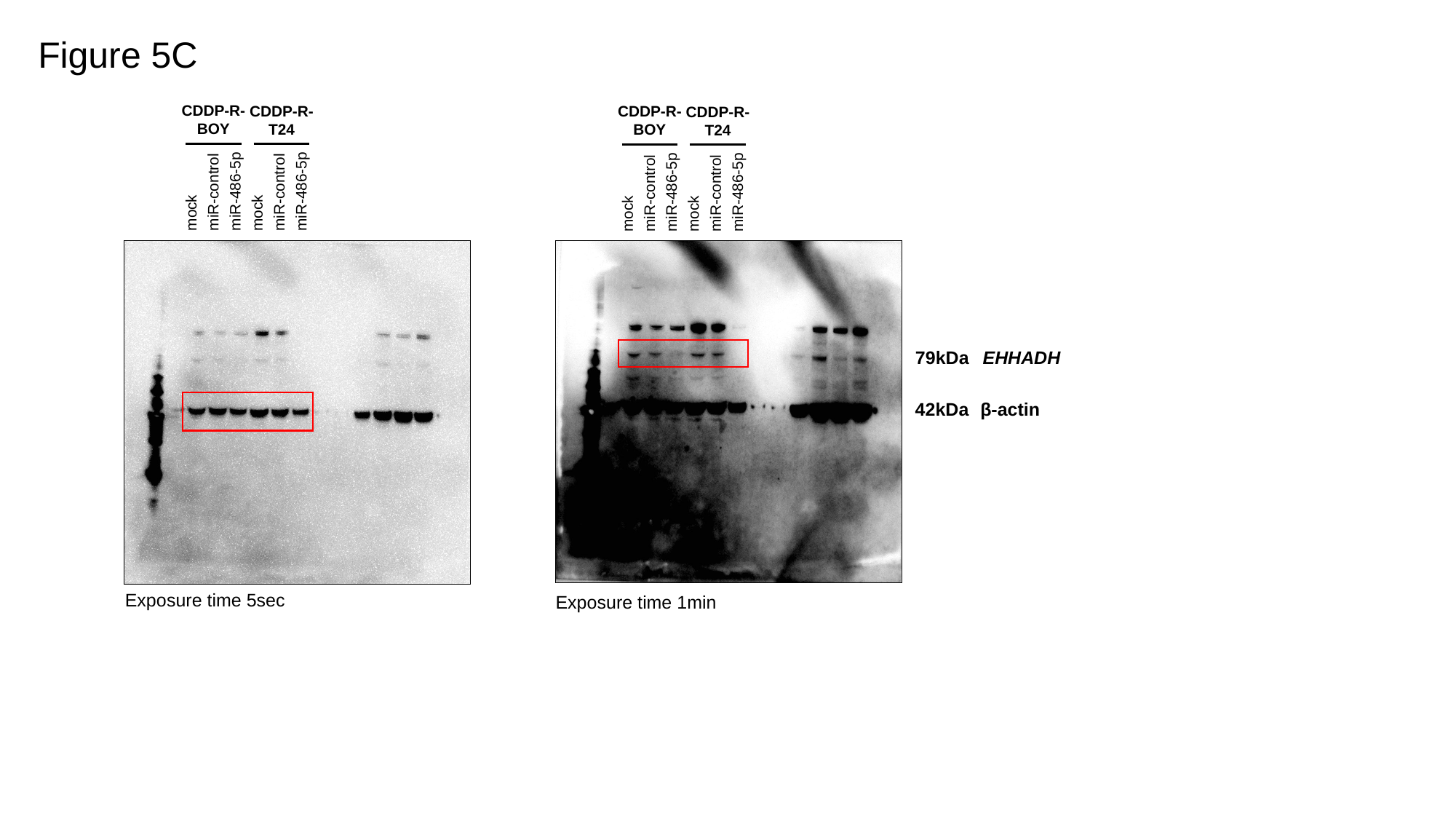

Figure 5C
CDDP-R-
BOY
CDDP-R-
T24
mock
miR-control
miR-486-5p
mock
miR-control
miR-486-5p
CDDP-R-
BOY
CDDP-R-
T24
mock
miR-control
miR-486-5p
mock
miR-control
miR-486-5p
79kDa
EHHADH
42kDa
β-actin
Exposure time 5sec
Exposure time 1min

## Slide 3
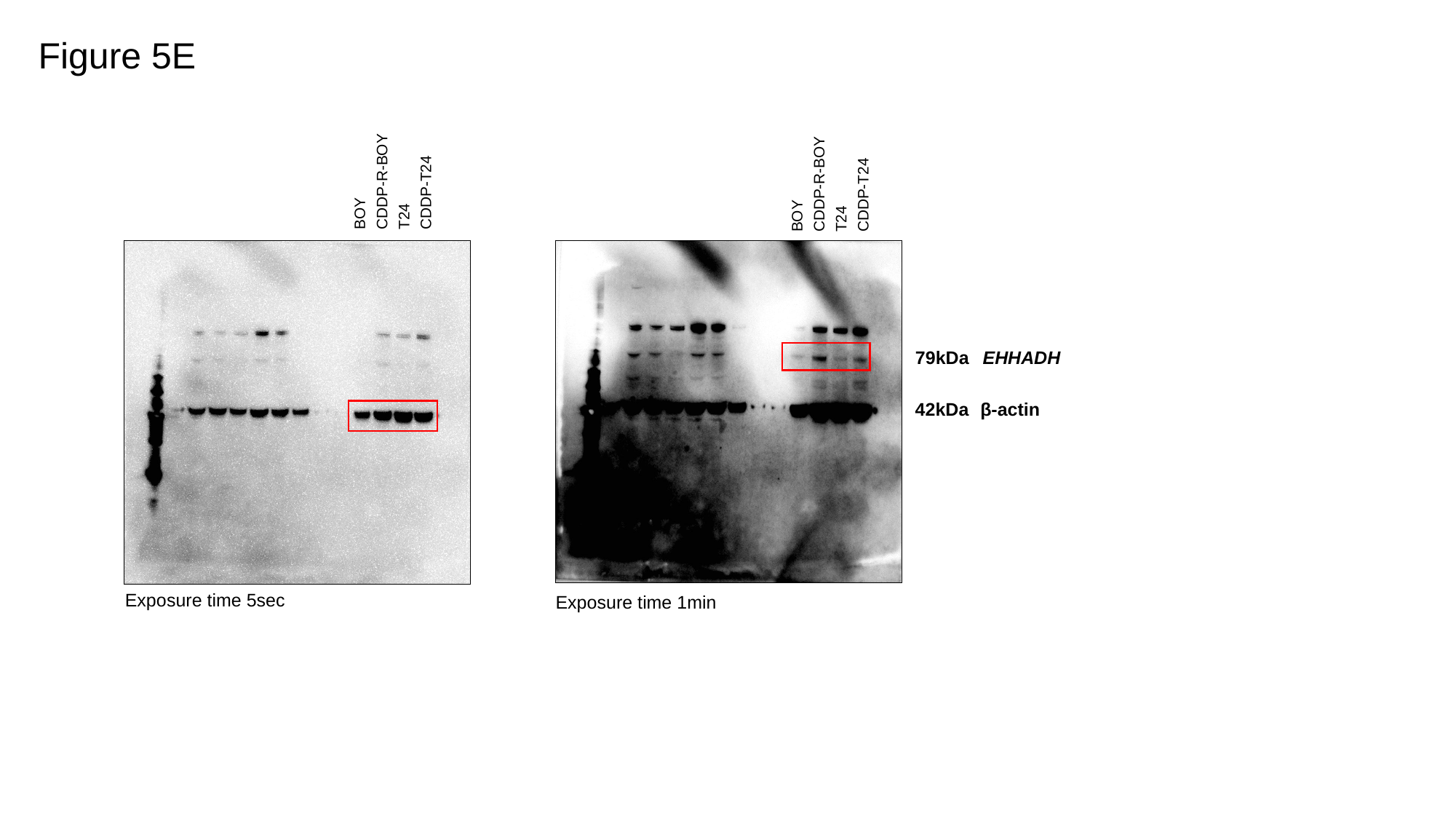

Figure 5E
BOY
CDDP-R-BOY
T24
CDDP-T24
BOY
CDDP-R-BOY
T24
CDDP-T24
79kDa
EHHADH
42kDa
β-actin
Exposure time 5sec
Exposure time 1min

## Slide 4
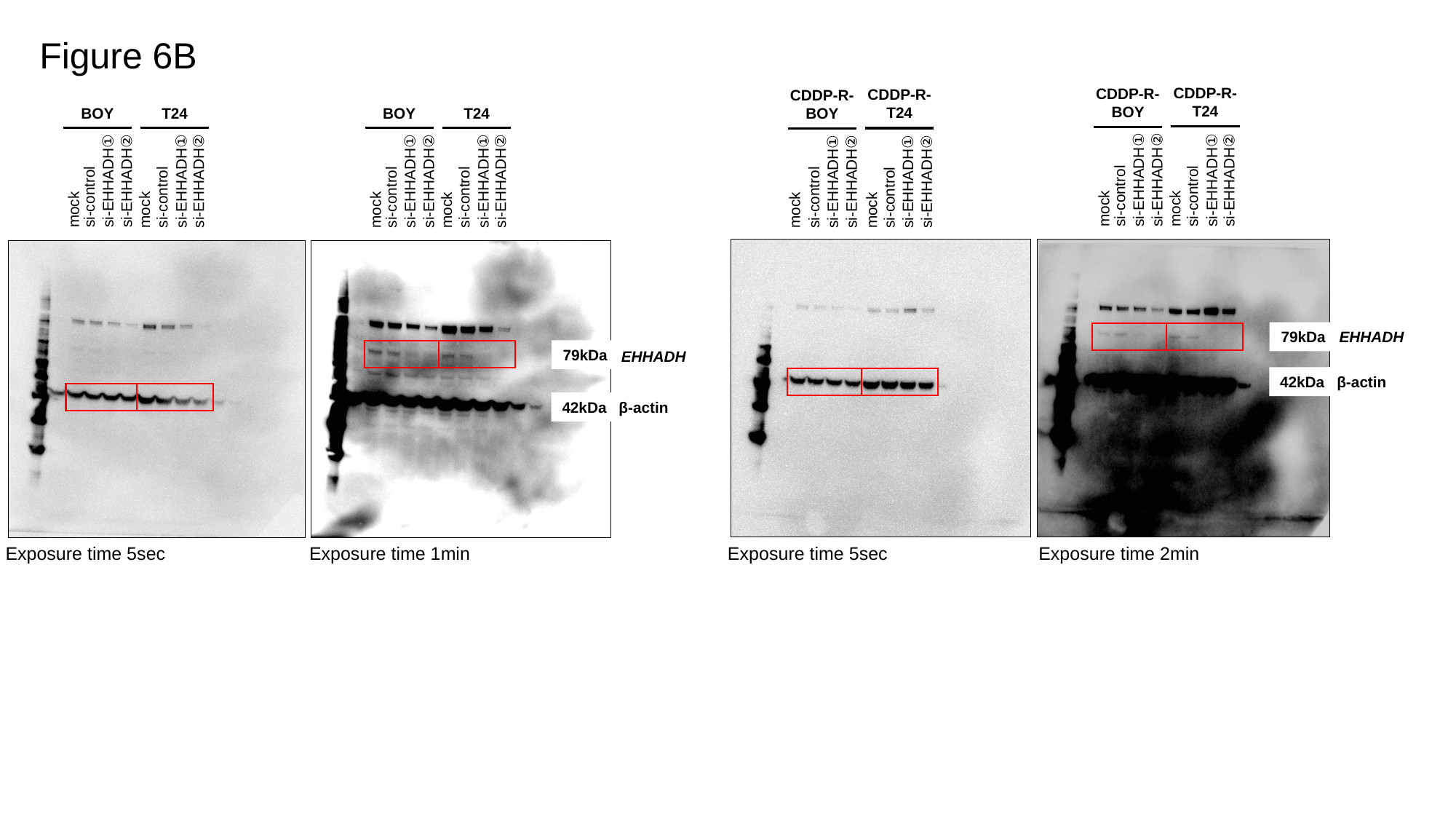

Figure 6B
CDDP-R-
T24
CDDP-R-
BOY
CDDP-R-
T24
CDDP-R-
BOY
BOY
T24
BOY
T24
si-EHHADH②
si-EHHADH①
si-EHHADH②
si-EHHADH①
mock
mock
si-control
si-control
si-EHHADH②
si-EHHADH①
si-EHHADH②
si-EHHADH①
mock
mock
si-control
si-control
si-EHHADH②
si-EHHADH①
si-EHHADH②
si-EHHADH①
mock
mock
si-control
si-control
si-EHHADH②
si-EHHADH①
si-EHHADH②
si-EHHADH①
mock
mock
si-control
si-control
79kDa
EHHADH
79kDa
EHHADH
42kDa
β-actin
42kDa
β-actin
Exposure time 2min
Exposure time 5sec
Exposure time 1min
Exposure time 5sec

## Slide 5
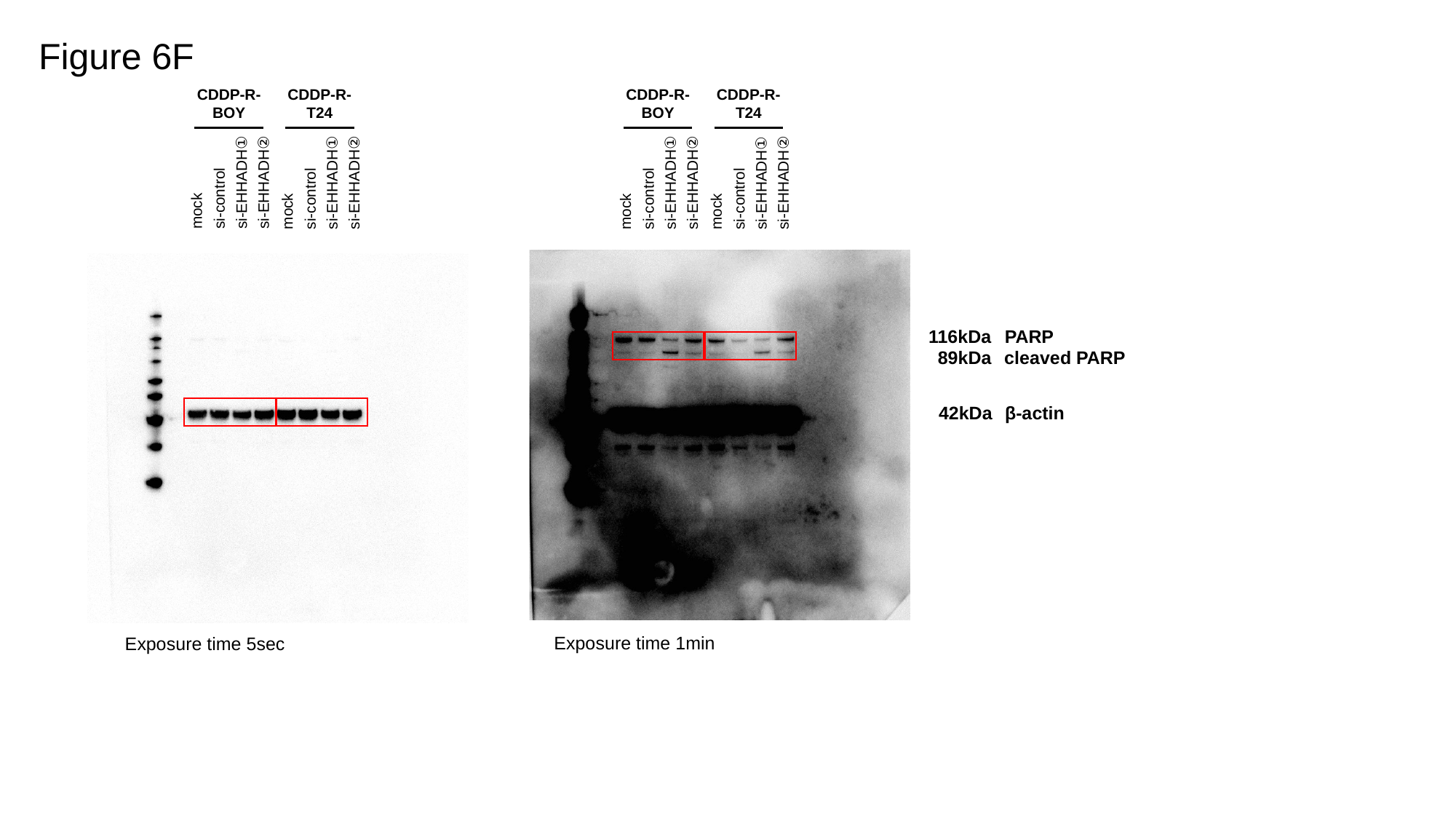

Figure 6F
CDDP-R-
BOY
CDDP-R-
BOY
CDDP-R-
T24
CDDP-R-
T24
si-EHHADH②
si-EHHADH①
si-EHHADH②
si-EHHADH①
si-EHHADH②
si-EHHADH①
si-EHHADH②
si-EHHADH①
mock
mock
mock
mock
si-control
si-control
si-control
si-control
116kDa
PARP
89kDa
cleaved PARP
42kDa
β-actin
Exposure time 1min
Exposure time 5sec
